# Supplementary material for: Features of acute COVID-19 associated with post-acute sequelae of SARS-CoV-2 phenotypes: results from the IMPACC study
Source: Nat Commun. 2024 Jan 3;15:216. doi: 10.1038/s41467-023-44090-5 (PMC10764789; doi:10.1038/s41467-023-44090-5)
Supplement: Supplementary file 1 — Supplementary Information [file 41467_2023_44090_MOESM1_ESM.pdf]

Features of acute COVID-19 associated with Post-Acute Sequelae of SARS-CoV-2 phenotypes: results from the IMPACC study

SUPPLEMENTAL MATERIALS

Supplementary Results

Of note, there were a total of 5 participants receiving anti-CD20 medications among the n=590 participants in this convalescent cohort i.e. less than 1%. These participants were distributed across several convalescent clusters (n=2 MIN, n=1 PHY, n=2 COG), all of which received rituximab. No participants received any other anti-CD20 medications.

Supplementary Methods

SARS-CoV-2 PCR

To assess SARS-CoV-2 viral load, nasal swabs were collected, and placed in 1ml of Zymo-DNA/RNA shield reagent (Zymo Research). RNA was extracted using the quick DNA-RNA MagBead kit (Zymo Research) following the manufacturer's instructions. RT-PCR for SARS-CoV-2 was performed on RNA extracts using the SARS-CoV-2 (2019-nCoV) CDC qPCR Probe Assay with target genes 2019-nCoV_N1, 2019-nCoV_N2, and control human RNase P (Integrated DNA Technologies). A ceiling cutoff of 40 cycles was applied as the limit of detection. Samples with an undetectable human RNase P were considered collection failures and were excluded.

Anti-SARS-CoV-2 Spike (S), and receptor binding domain (RBD) antibodies

Readouts were generated for serology measures, endpoint titers, and area under the curve (AUC) values in duplicates. Area Under the Curve (AUC) considers not only the endpoint titer of ELISA curves but also the magnitude of the signal at each dilution, and therefore provides more granularity. For this paper, AUC was chosen as the main readout.

Autoantibodies

Serum samples were screened at the baseline visit, for autoantibodies against type I IFNs in a multiplex, particle-based assay, in which differentially fluorescing magnetic beads were covalently coupled to recombinant human proteins (2.5 mcg/reaction)[1]. Beads were combined and incubated with 1:100 diluted serum samples for 30 minutes. Each sample was tested once with a random assortment in each plate tested in duplicate to ensure minimal intra-assay variability. Beads were washed then incubated with PE-labeled goat anti-human IgG (1µg/mL) for an additional 30 minutes. Beads were washed again and run on a BioPlex X200 instrument in a multiplex assay. Participant samples with a fluorescence intensity greater than 3 standard deviations above the mean of 1099 healthy controls at the earliest timepoint received (>1310 FI for IFNa; > 386 FI for IFNb; > 1387 for IFNw) were tested for blocking activity in the pSTAT1 functional assay,

pSTAT1 functional assay

The blocking activity of anti-IFN-containing serum was determined by assessing STAT1 phosphorylation in healthy control cells following stimulation with the appropriate cytokine in the presence of 10% healthy control or participant serum[1]. Surface-stained healthy control PBMCs (350,000/reaction) were cultured in serum-free RPMI medium with 10% healthy control (pooled human AB serum) or participant serum and were either left unstimulated or stimulated with IFNa, IFNw, or IFNb (10 ng/mL) for 15 minutes at 37°C. Cells were fixed, permeabilized, and stained for intranuclear phospho-STAT1 (Y701). Cells were acquired on a BD LSRFortessa cytometer with gating on CD14+ monocytes and analyzed with FlowJo software version 10.9.0. A stimulation index was calculated for each sample by dividing the geometric mean fluorescence for the pSTAT1 channel for each stimulated condition by that of the unstimulated condition. The stimulation index for each cytokine was then normalized to that of the healthy control serum from the same assay, generating a normalized stimulation index (where >65% pSTAT1 = NOT blocking; 20-65% pSTAT1 = partially blocking; <20% pSTAT1 = blocking).

<u>CyTOF</u>

Blood samples were collected at study time points and processed within 6 hours for multiparameter CyTOF[2]. Whole blood anticoagulated with EDTA (270 µl) was labeled at room temperature for 30 min with a 30-marker Maxpar Direct Immune Profiling Assay (Standard Biotools, South San Francisco, CA) panels, fixed and cryopreserved using PROT-1 proteomic stabilizer (SMART, Inc, Las Vegas, NV) and frozen at -80˚C. Batches of samples were randomized by study site, disease severity (mild/moderate versus severe), and age (younger versus older) and thawed, washed, and labeled with a supplemental cocktail of 14 additional antibodies to identify additional fixation-resistant cell phenotype determinants. Labeled samples were resuspended to 7-8 x 10^5/mL in CAS or MilliQ containing a ten-fold dilution of Fluidigm EQ 4-Element normalization beads and acquired on the Fluidigm Helios mass cytometer in multiple acquisitions with approximately 250-350 events/sec acquisition rate. On the day of acquisition, the Helios instrument was tuned according to manufacturer's software standards with calibration of Tb159 or Tm169 signal from the Fluidigm Tuning Solution to be within 10% across batches. Pooled samples were acquired until a total of 6 x 10^6 cell events had been collected, corresponding to an average target event number of 3 x10^5 events per original donor subsample. The resulting FCS files are evaluated using a centralized data processing pipeline including bead-based sample QC and data normalization and automated sample demultiplexing[2]. The analysis pipeline QC removed multiplets based on the Gaussian parameters Residual and Offset acquired by the Helios mass cytometer. Immune cell frequencies as a percentage of CD45+ immune cells (excluding debris, RBCs, platelets, and multiplets) were determined from K-means clusters with cell identities assigned in Clustergrammer2 based on a manually annotated training dataset to define cell population frequencies.

To account for differences in cell events acquired for each sample, the cell population count matrix was converted into cell frequencies. We first processed immune cell frequencies of the more broadly defined cell subsets (e.g.,CD4 T cells, B cells, monocytes, etc.) as a percentage of total CD45+ immune cells by excluding

debris, RBCs, platelets, and multiplets. Next, we processed the broadly defined cell subsets as a percentage of all non-granulocytes by further excluding eosinophils, neutrophils, and basophils. We also processed immune cell frequencies of the more granularly defined subsets (e.g.,CD4 effector memory T cells, Naive B cells, CD14+CD16- classical monocytes, etc.) as a percentage of total CD45+ immune cells and as a percentage of non-granulocytes.

Proximity Extension Assay (O-Link)

Study samples were assayed in plate batch layouts following a centralized randomized scheme described above. All sera were subjected to Olink multiplex assay Inflammatory panel (Olink Bioscience, Uppsala, Sweden), according to the manufacturer's instructions. This inflammatory panel included 92 proteins associated with human inflammatory conditions. An incubation master mix containing pairs of oligonucleotide-labeled antibodies to each protein was added to the samples and incubated for 16 hours at 4 ºC. Each protein was targeted with two different epitope-specific antibodies, increasing the assay's specificity. The presence of the target protein in the sample brought the partner probes in close proximity, allowing the formation of a double-strand oligonucleotide polymerase chain reaction (PCR) target. On the following day, the extension master mix in the sample initiated the specific target sequences to be detected and generated amplicons using PCR in 96 well plates. For the detection of the specific protein, Dynamic array integrated fluidic Circuit (IFC) 96x96 chip was primed, loaded with 92 protein-specific primers, and mixed with sample amplicons, including three inter-plate controls (IPS) and three negative controls (NC). Real-time microfluidic qPCR was performed in Biomark (Fluidigm, San Francisco, CA) for the target protein quantification.

Plasma global metabolomics

The study conducted plasma metabolite profiling using Metabolon's solvent extraction method and in-house standards[3]. Samples were divided into randomized batches, extracted, and prepared for analysis with the use of recovery standards for quality control. Protein was removed by methanol precipitation and centrifugation. The supernatants were analyzed using multiple reverse phase/UPLC-MS/MS methods with positive and negative ion mode electrospray ionization, HILIC/UPLC-MS/MS with negative ion mode ESI and a backup analysis was done using Waters ACQUITY UPLC and a Thermo Scientific Q-Exactive mass spectrometer. Parameters for metabolomics run and solvents used are provided[4]. Metabolites were identified by comparison to Metabolon's library of standard metabolites based on retention index, accurate mass, and MS/MS scores. Compounds were categorized according to the Metabolomics Standards Initiative's guidelines and orthogonal analytical techniques were applied for confirmation[3]. Metabolites with accurate mass confirmed via MS, retention index, chemical, and composition were reported.

Plasma global metabolomics spectral raw data were reported based on LC-MS peak areas proportional to metabolite feature concentration. Any missing values in the dataset were imputed with half the minimum detected level for a given metabolite. Additionally, metabolites with an interquartile range of 0 were excluded from the

subsequent analysis. Features were log-transformed, normalized and Pareto-scaled to reduce the variation in fold change differences between features. The analyses of the processed data utilized the Weighted Gene Co-expression Network Analysis (WGCNA) to identify modules of co-expressed metabolites. In addition, we performed generalized linear mixed effects models (GLM) and generalized additive mixed models (GAM) (as described) to explore individual associations between metabolite modules and PRO outcomes.

Table 1A-S: Comparison across minimal deficit clusters (MIN). For each PRO, t-statistics comparing within-cluster means versus all remaining clusters and pairwise comparisons between each cluster. T-statistics are recoded such that negative values indicate a greater degree of patient-reported deficit. t-statistics and two-sided p-values from two-sample t-tests. P-values from pairwise comparisons are not adjusted for multiple comparisons.

	**t-statistic (p-value)**<br>**Collapsed into MIN (n=358)**			**Pairwise comparisons**<br>*p<0.05 **p<0.01 ***p<0.001		
	**Cluster A (n=109)**	**Cluster B (n=119)**	**Cluster C (n=130)**	**A vs. B**	**A vs. C**	**B vs. C**
**Health**	10.74 (<0.001)	2.46 (0.015)	4.50 (<0.001)	***	***	
**EQ-5D-5L**	14.36 (<0.001)	4.80 (<0.001)	6.10 (<0.001)	***	***	
**Physical**	14.67 (<0.001)	1.96 (0.052)	5.37 (<0.001)	***	***	
**Dyspnea**	8.44 (<0.001)	2.89 (0.004)	1.12 (0.27)	**	***	
**Mental**	24.86 (<0.001)	5.80 (<0.001)	4.19 (<0.001)	***	***	*
**Psychosocial Impact**	15.32 (<0.001)	27.41 (<0.001)	-5.43 (<0.001)	***	***	***
**Cognitive**	10.95 (<0.001)	3.08 (0.003)	0.48 (0.63)	***	***	

Table 1B-S: For each PRO, t-statistics comparing within-cluster means versus remaining clusters and pairwise comparisons between each cluster. T-statistics are recoded such that negative values indicate a greater degree of patient-reported deficit. Clusters: minimal deficit (MIN); physical predominant deficit (PHY); mental/cognitive predominant deficit (COG); and multidomain deficit (MLT). t-statistics and two-sided p-values from two-sample t-tests. P-values from pairwise comparisons are not adjusted for multiple comparisons.

	**t-statistic (p-value)**				**Pairwise comparisons** *p<0.05 **p<0.01 ***p<0.001					
	**MIN (n=358)**	**PHY (n=92)**	**COG (n=82)**	**MLT (n=58)**	**MIN vs. PHY**	**MIN vs. COG**	**MIN vs. MLT**	**PHY vs. COG**	**PHY vs. MLT**	**COG vs. MLT**
**Health**	12.75 (<0.001)	-2.69 (0.008)	-5.59 (<0.001)	-13.72 (<0.001)	***	***	***		***	***
**EQ-5D-5L**	17.40 (<0.001)	-5.33 (<0.001)	-6.33 (<0.001)	-18.01 (<0.001)	***	***	***		***	***
**Physical**	17.87 (<0.001)	-11.93 (<0.001)	-4.31 (<0.001)	-15.64 (<0.001)	***	***	***	***	*	***
**Dyspnea**	7.90 (<0.001)	-2.84 (0.005)	-3.13 (0.002)	-5.12 (<0.001)	***	***	***			
**Mental**	23.79 (<0.001)	-4.94 (<0.001)	-18.50 (<0.001)	-21.88 (<0.001)	***	***	***	***	***	***
**Psychosocial Impact**	18.82 (<0.001)	-1.00 (0.32)	-17.04 (<0.001)	-16.73 (<0.001)	***	***	***	***	***	***
**Cognitive**	9.45 (<0.001)	-3.01 (0.003)	-8.80 (<0.001)	-3.36 (0.001)	***	***	***	*		

Table 2S: Participants with autoantibodies against interferon (IFN) alpha, beta and omega by age, sex and patient reported outcome (PRO) clusters. P-values are computed using Fisher's exact test and P-values are two sided.

	Total number of participants tested	Total number of participants with autoantibodies (percentage)	p-value
Sex			0.14
Female	221	6 (2.7%)	
Male	342	18 (5.3%)	
Age			0.04
[19-35]	56	1 (4%)	
[36-50]	152	4 (2.8%)	
[51-65]	220	7 (2.4%)	
[66-80]	121	10 (10%)	
[81-95]	14	2 (12%)	
PRO cluster			0.20
Minimal deficit (MIN)	341	11 (3.2%)	
Physical predominant deficit (PHY)	87	6 (6.9%)	
Mental/cognitive predominant deficit (COG)	80	3 (3.8%)	
Multidomain deficit (MLT)	55	4 (7.3%)	

Figure 1S: Overlap in convalescent symptoms reported within organ class at least once during convalescence period (up to 12 months after hospital discharge).

Sx=symptoms and any other sx= gastrointestinal, upper respiratory or neurologic symptoms

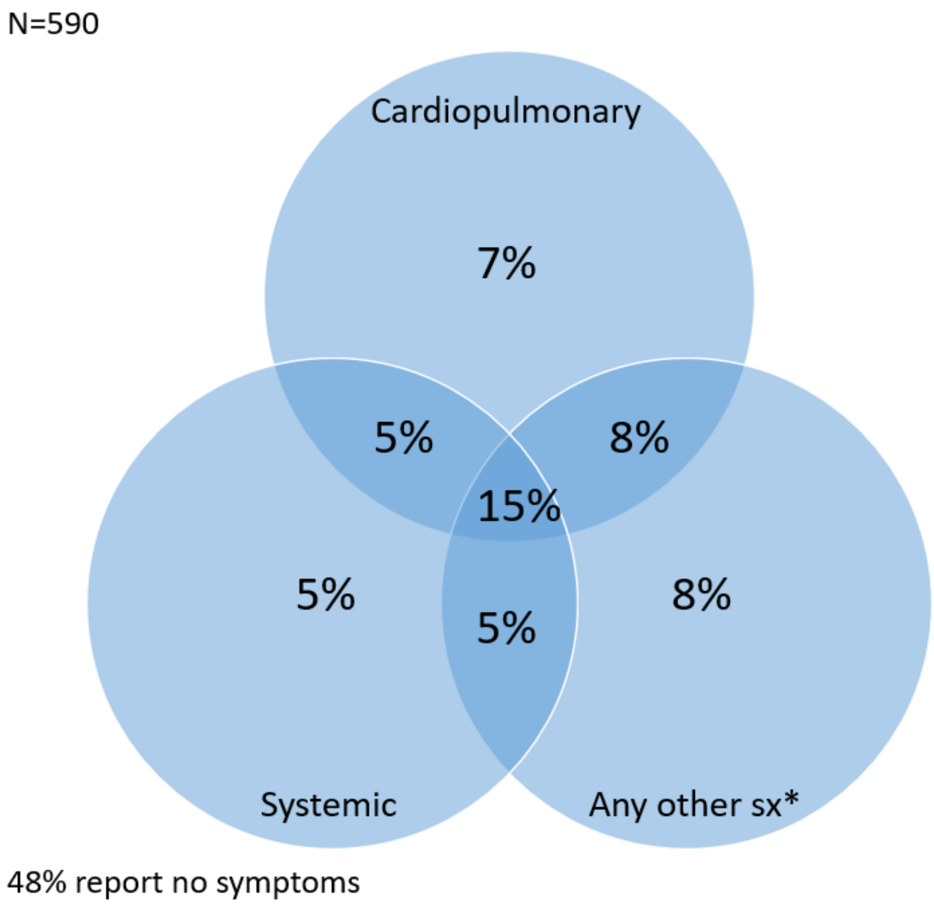

Figure 2S: Frequency of symptoms at hospital admission and during convalescent period

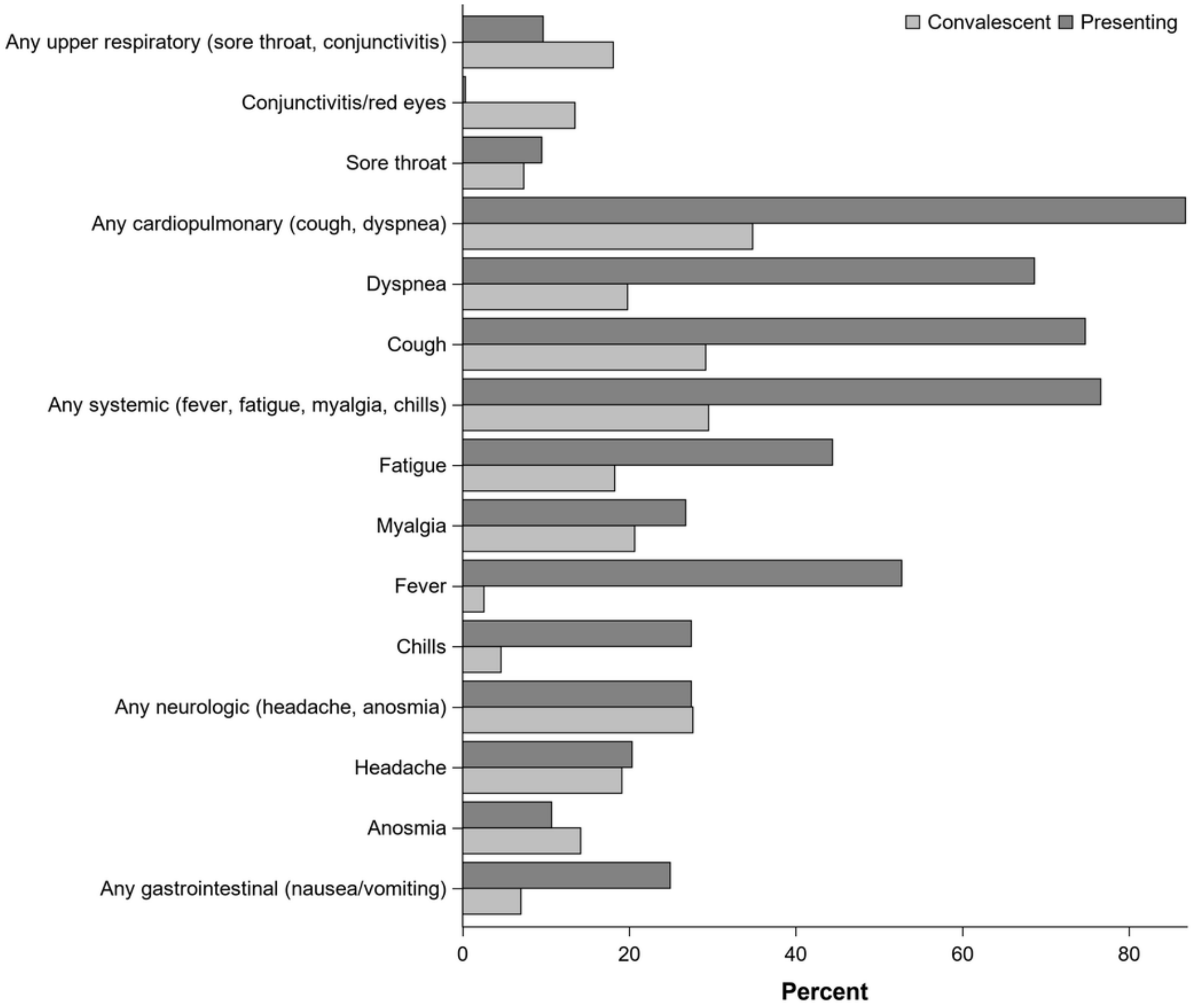

Figure 3S: Final groupings for each Patient Reported Outcome (PRO) over four longitudinal timepoints. Solid line denotes within-group median, shading denotes IQR.

A: EQ-5D-5L; 283 (48.3%) High, 204 (34.8%) Medium, 99 (16.9%) Low, n=4 missing

B: Health Recovery score; 260 (44.3%) High, 205 (35.0%) Medium, 121 (20.7%) Low, n=4 missing

C: PROMIS Cognitive Function score; 180 (40.1%) High, 97 (21.7%) Medium, 171 (38.2%) Low, n=142 missing

D: PROMIS Dyspnea score; 127 (21.7%) High, 107 (18.3%) Medium, 352 (60.0%) Low, n=4 missing

E: PROMIS Physical Function score; overall median=44.9 (IQR=38.2, 52.5)

F: PROMIS Global Mental Health score; overall median=50.0 (IQR=44.4, 56.9)

G: PROMIS Psychosocial Illness Impact Positive score; overall median=50.8 (IQR=43.4, 57.8)

H: Clustering fitting statistics for 2-10 clusters across five clustering algorithms.

Panels from left to right show the Dunn index, average silhouette width, ratio of within to between sums of squares (SS), and within-cluster SS.

A: EQ-5D-5L

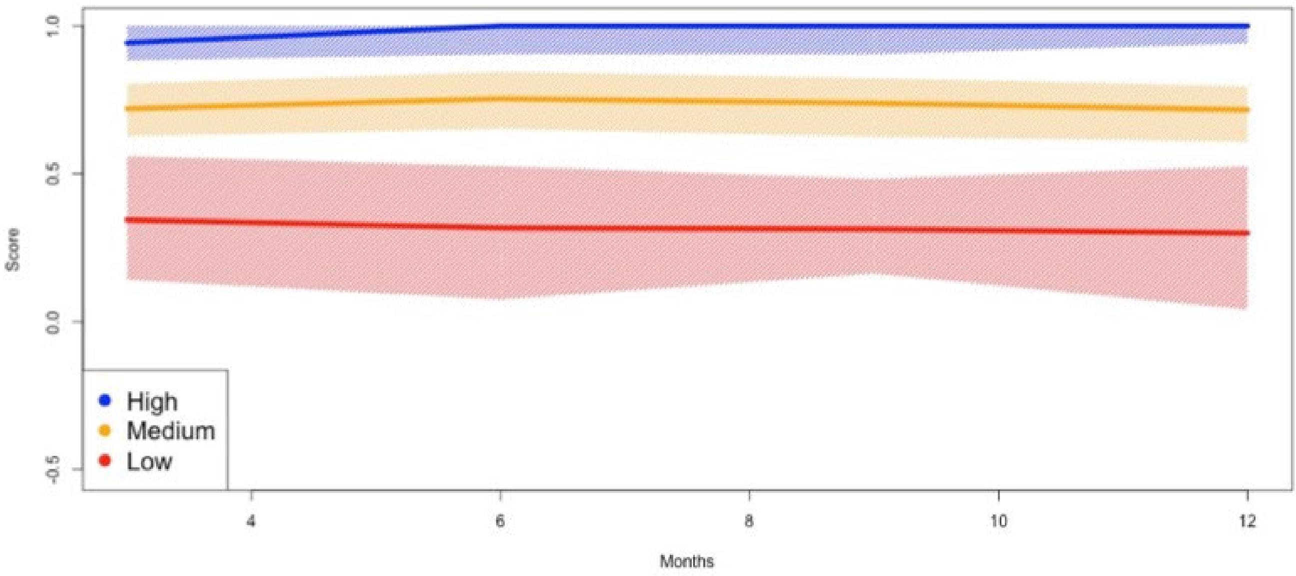

B: Health Recovery Score

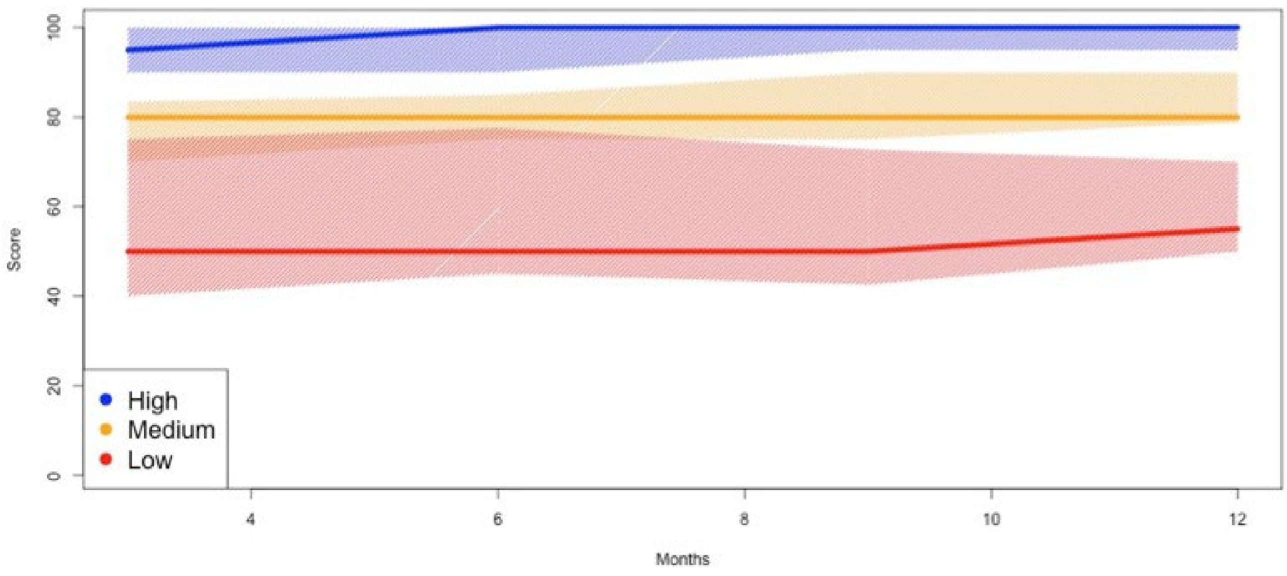

C: PROMIS Cognitive Function Score

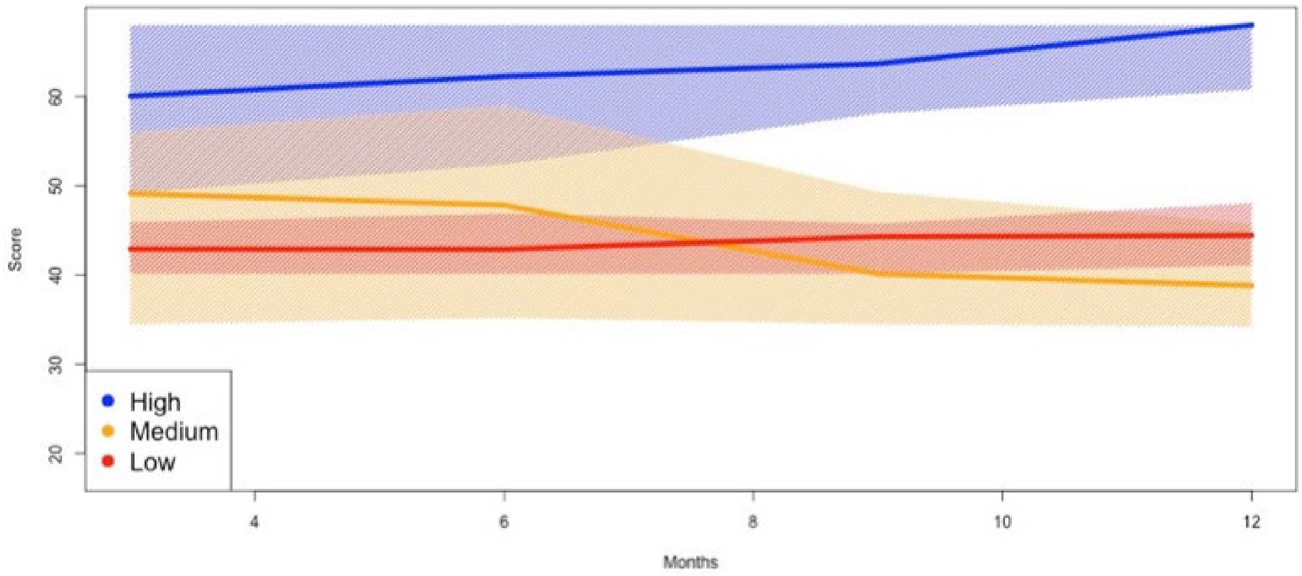

D: PROMIS Dyspnea score

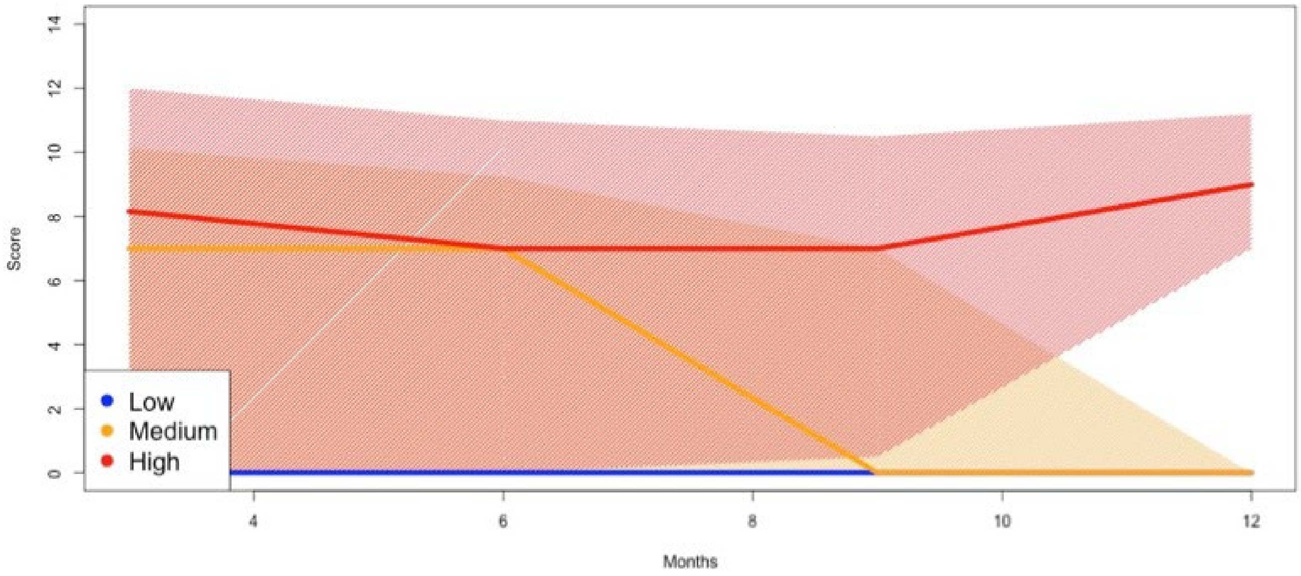

E: PROMIS Physical Function score

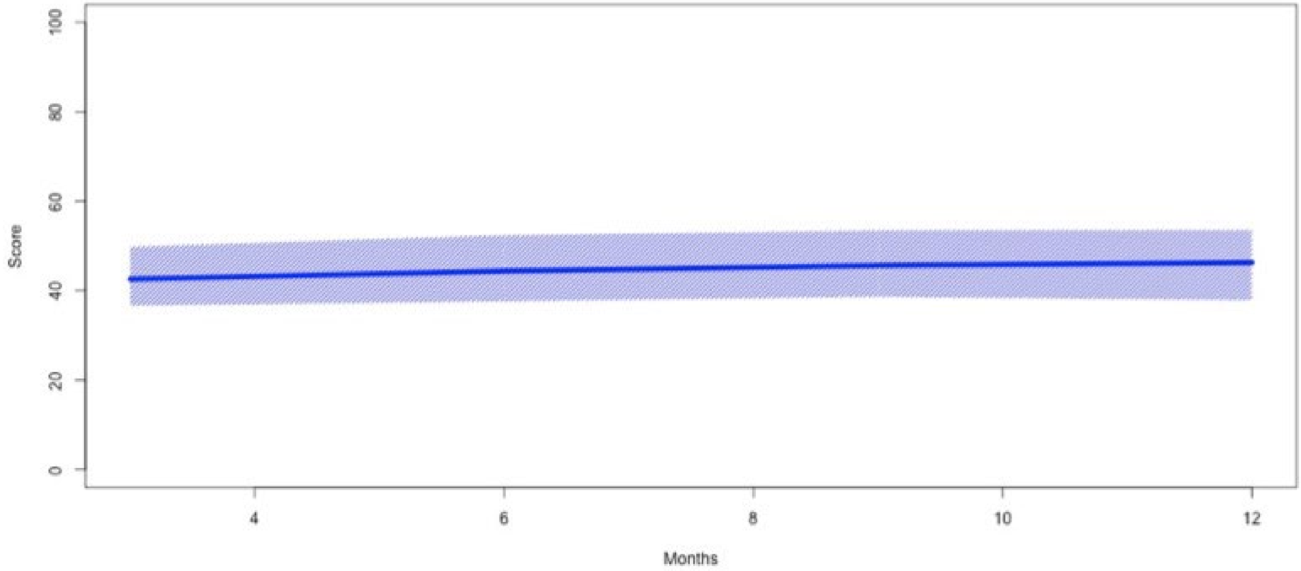

F: PROMIS Global Mental Health score

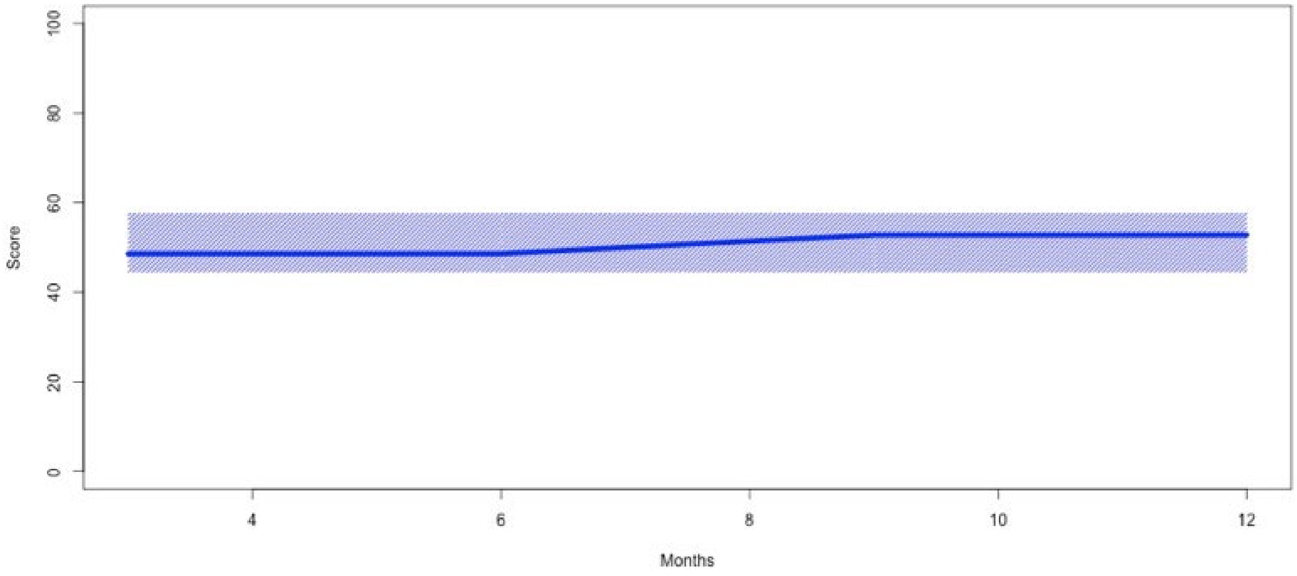

G: PROMIS Psychosocial Illness Impact Positive score

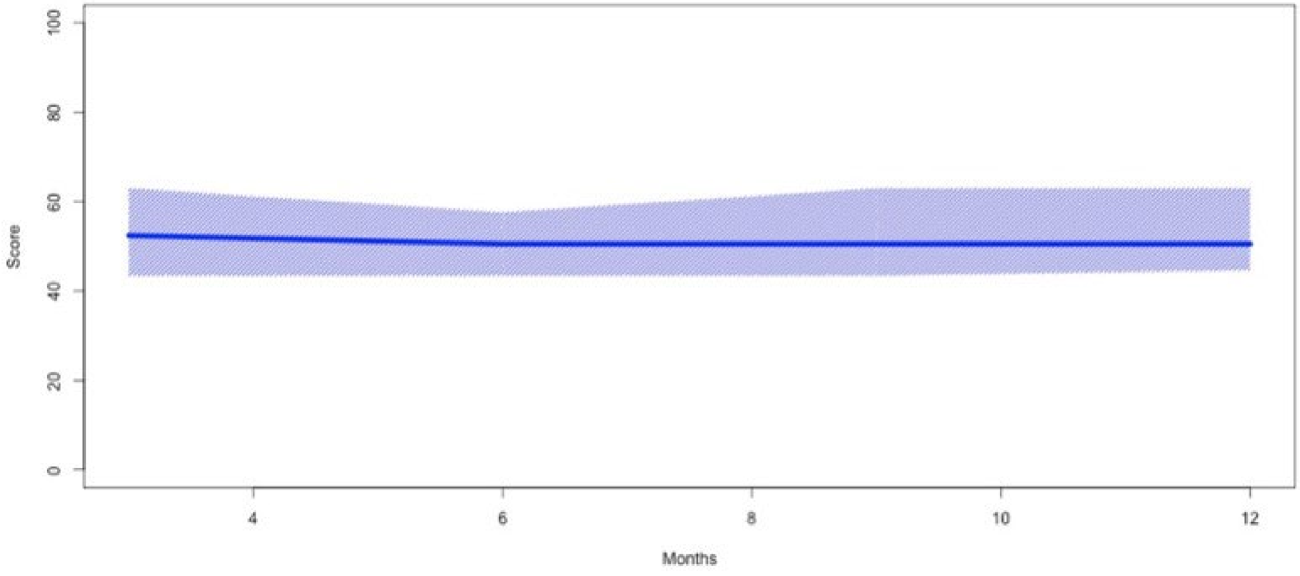

H: Cluster fitting statistics

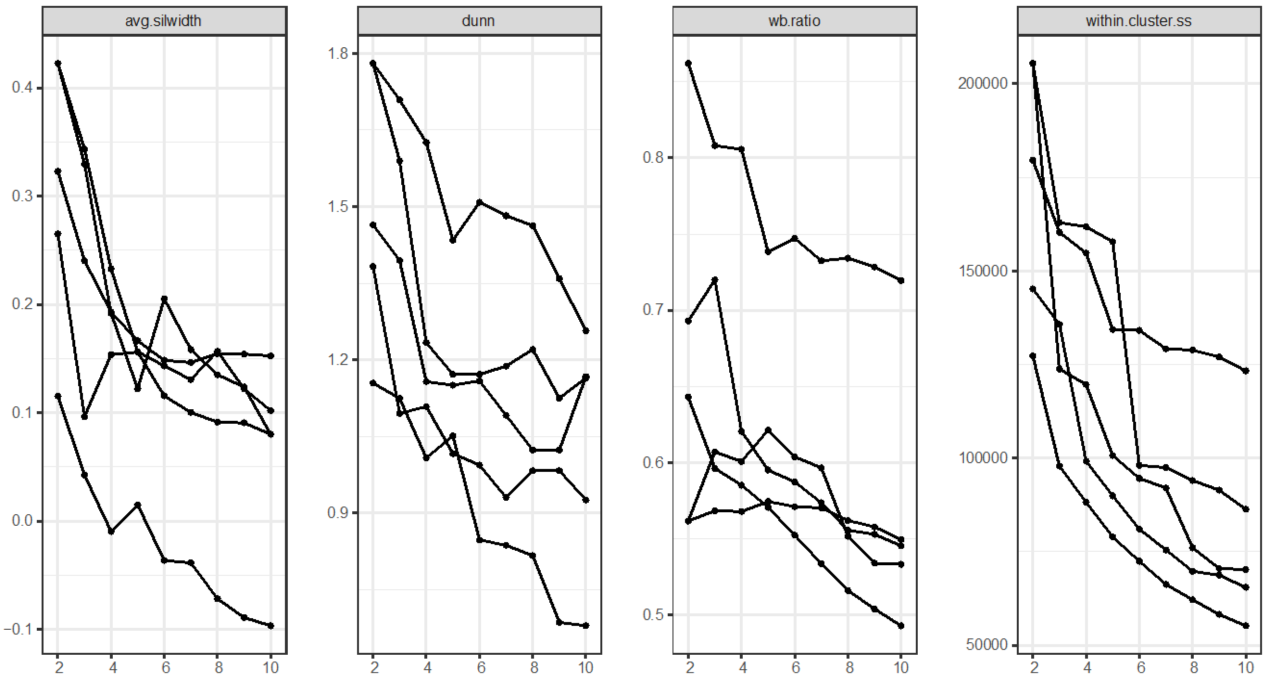

Figure 4S:

A- N2 Ct values: Shown are SARS-CoV-2 N2 gene PCR cycle threshold (Ct) values (viral loads) measured from samples collected during the first 28 days of hospital admission by four PRO clusters, minimal deficit (MIN, n= 657), physical predominant (PHY, n = 174), deficit, mental/cognitive predominant (COG, n = 172) and deficit, multidomain (MLT, n = 112). Shown are median values (horizontal lines), interquartile ranges (boxes), and 1.5 IQR (whiskers), as well as all individual points. Because lower Ct values indicate higher viral loads, the y-axis is reversed. The viral loads were significantly (adj. p= 0.017) associated with the PRO clusters. Statistical differences were determined from generalized linear mixed effects models adjusting for age, sex, participant and enrollment site, see Methods for more details.

B- Anti-Spike IgG values: Shown are anti-Spike IgG values measured from samples collected during the first 28 days of hospital admission by four PRO clusters, minimal deficit (MIN, n= 907), physical predominant (PHY, n = 221), deficit, mental/cognitive predominant (COG, n = 230) and deficit, multidomain (MLT, n = 149). Shown are median values of area under the curve (horizontal lines), interquartile ranges (boxes), and 1.5 IQR (whiskers), as well as all individual points. The titers were significantly (adj. p= 0.014) associated with the PRO clusters. Statistical differences were determined from generalized linear mixed effects models adjusting for age, sex, participant and enrollment site, see Methods for more details.

C- Ratio of anti-Spike IgG to N1 values: Shown are scaled ratio of anti-Spike IgG to SARS-CoV-2 viral loads (N1 gene) values from samples collected during the first 28 days of hospital admission by four PRO clusters, minimal deficit (MIN, n= 560), physical predominant (PHY, n = 156), deficit, mental/cognitive predominant (COG, n = 141) and deficit, multidomain (MLT, n = 99). Shown are median values (horizontal lines), interquartile ranges (boxes), and 1.5 IQR (whiskers), as well as all individual points. The ratio of titers to viral loads were also significantly (adj. p= 0.05) associated with the PRO clusters. Statistical differences were determined from generalized linear mixed effects models adjusting for age, sex, participant and enrollment site, see Methods for more details.

D- N1 Ct values over time: Shown are the SARS-CoV-2 N1 gene Ct values collected during the first 28 days of hospital admission by four PRO clusters, minimal deficit (MIN, n= 600), physical predominant (PHY, n = 162), deficit, mental/cognitive predominant (COG, n = 157) and deficit, multidomain (MLT, n = 105). The points represent the individual Ct values. The solid black trend line for each PRO cluster represents the fit of a generalized additive model (GAM) to capture non-linear trends in the data. The grey lines represent individual fitted lines for each participant. The viral loads were significantly (shape adj. p= 0.3, average adj.

p= 0.015) associated with the PRO clusters. Statistical differences were determined from generalized additive mixed effects models adjusting for age, sex, participant and enrollment site, see Methods for more details.

E- N2 Ct values over time: Shown are the SARS-CoV-2 N1 gene Ct values collected during the first 28 days of hospital admission by four PRO clusters, minimal deficit (MIN, n= 600), physical predominant (PHY, n = 162), deficit, mental/cognitive predominant (COG, n = 157) and deficit, multidomain (MLT, n = 105). The points represent the individual Ct values. The solid black trend line for each PRO cluster represents the fit of a generalized additive model (GAM) to capture non-linear trends in the data. The grey lines represent individual fitted lines for each participant. The viral loads were significantly (shape adj. p= 0.3, average adj. p= 0.013) associated with the PRO clusters. Statistical differences were determined from generalized additive mixed effects models adjusting for age, sex, participant and enrollment site, see Methods for more details.

F- Anti-RBD IgG over time: Shown are the anti-RBD IgG values (AUC) collected during the first 28 days of hospital admission by four PRO clusters, minimal deficit (MIN, n= 810), physical predominant (PHY, n = 199), deficit, mental/cognitive predominant (COG, n = 206) and deficit, multidomain (MLT, n = 137). The points represent the individual ratio values. The solid black trend line for each PRO cluster represents the fit of a generalized additive model (GAM) to capture non-linear trends in the data. The grey lines represent individual fitted lines for each participant. The titers were significantly (shape adj. p= 0.005, average adj. p= 0.023) associated with the PRO clusters. Statistical differences were determined from generalized additive mixed effects models adjusting for age, sex, participant and enrollment site, see Methods for more details.

G- Anti-Spike IgG over time: Shown are the anti-Spike IgG values (AUC) collected during the first 28 days of hospital admission by four PRO clusters, minimal deficit (MIN, n= 810), physical predominant (PHY, n = 199), deficit, mental/cognitive predominant (COG, n = 206) and deficit, multidomain (MLT, n = 137). The points represent the individual ratio values. The solid black trend line for each PRO cluster represents the fit of a generalized additive model (GAM) to capture non-linear trends in the data. The grey lines represent individual fitted lines for each participant. The titers were significantly (shape adj. p= 0.0017, average adj. p= 0.023) associated with the PRO clusters. Statistical differences were determined from generalized additive mixed effects models adjusting for age, sex, participant and enrollment site, see Methods for more details.

H- Ratio of anti-RBD IgG to N1 over time: Shown are the trends of the scaled ratio of anti-RBD IgG values divided by SARS-CoV-2 viral loads (N1 gene) for time points collected during the first 28 days of hospital admission by four PRO clusters, minimal deficit (MIN, n= 517), physical predominant (PHY, n = 145), deficit,

mental/cognitive predominant (COG, n = 132) and deficit, multidomain (MLT, n = 94). The ratio of titers to viral loads were also significantly (shape adj. p= 0.094, average adj. p= 0.009) associated with the PRO clusters. Statistical differences were determined from generalized additive mixed effects models adjusting for age, sex, participant and enrollment site, see Methods for more details.

I- Ratio of anti-Spike IgG to N1 over time: Shown are the trends of the scaled ratio of anti-Spike IgG values divided by SARS-CoV-2 viral loads (N1 gene) for time points collected during the first 28 days of hospital admission by four PRO clusters, minimal deficit (MIN, n= 517), physical predominant (PHY, n = 145), deficit, mental/cognitive predominant (COG, n = 132) and deficit, multidomain (MLT, n = 94). The ratio of titers to viral loads were also significantly (shape adj. p= 0.094, average adj. p= 0.02) associated with the PRO clusters. Statistical differences were determined from generalized additive mixed effects models adjusting for age, sex, participant and enrollment site, see Methods for more details.

The four PRO clusters are the following in grey: minimal deficit (MIN), in blue: deficit, physical predominant (PHY), in yellow: deficit, mental/cognitive predominant (COG) and in red: deficit, multidomain (MLT). The lines and asterisks on top of the figure denote pairwise statistical significance, * $p < 0.05$, ** $p < 0.01$, *** $p < 0.001$ and NS denote not significant. P-values were adjusted using the Benjamini-Hochberg method to account for multiple comparisons.

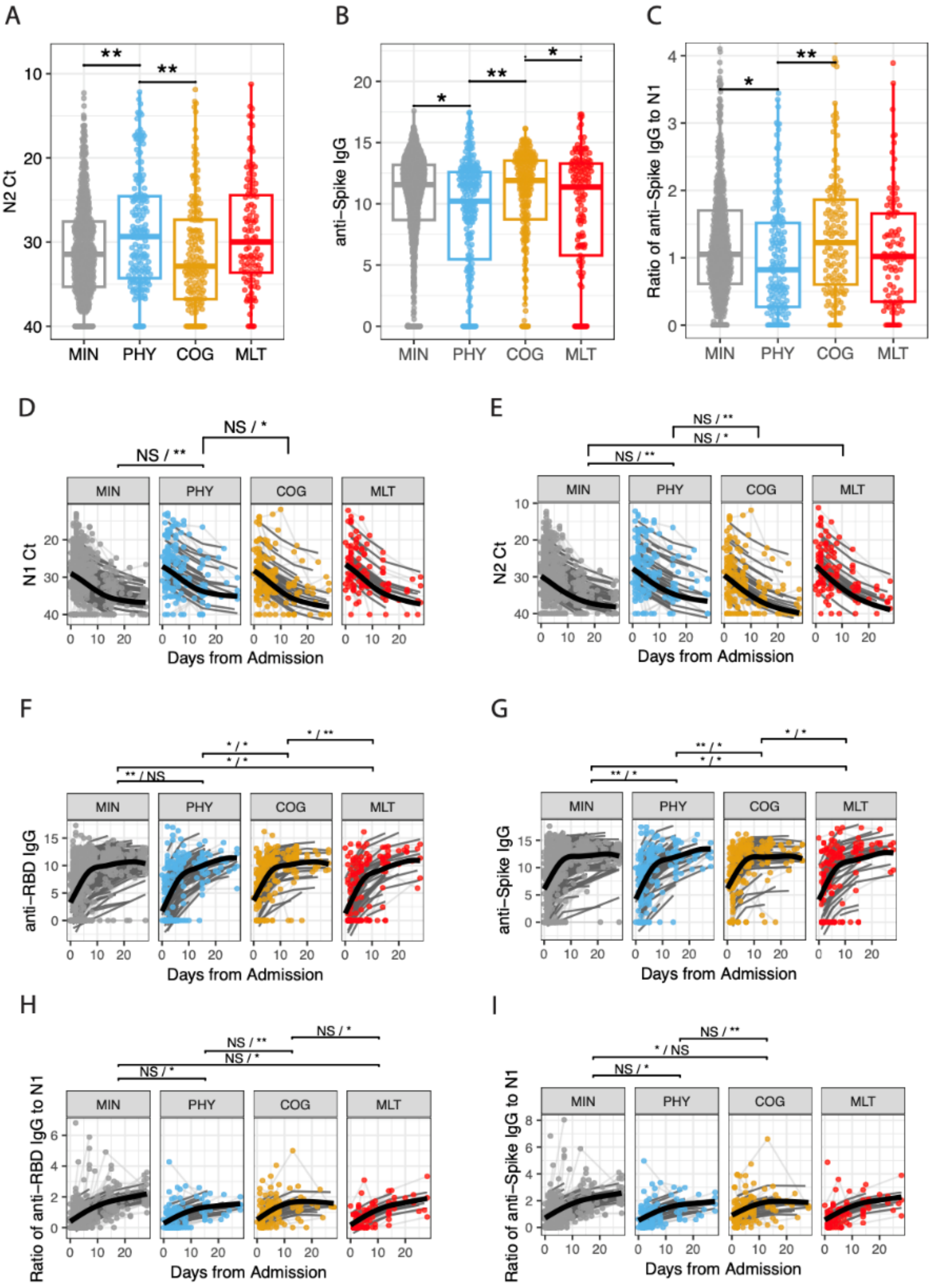

A
N2 Ct
MIN PHY COG MLT
B
anti-Spike IgG
C
Ratio of anti-Spike IgG to N1
D
NS / **
NS / *
N1 Ct
Days from Admission
E
N2 Ct
F
anti-RBD IgG
G
anti-Spike IgG
H
Ratio of anti-RBD IgG to N1
I
Ratio of anti-Spike IgG to N1

Figure 5S:

A-Naïve B cell to non-granulocyte frequency: Shown are aïve B cell to non-granulocyte frequency values from samples collected during the first 28 days of hospital admission by four PRO clusters, minimal deficit (MIN, n= 584), physical predominant (PHY, n = 140), deficit, mental/cognitive predominant (COG, n = 145) and deficit, multidomain (MLT, n = 107). Shown are median values (horizontal lines), interquartile ranges (boxes), and 1.5 IQR (whiskers), as well as all individual points. The repeated-measurement model did not show statistically significant association between the frequency and PRO clusters (adj. p= 0.2466). Statistical differences were determined from generalized linear mixed effects models adjusting for age, sex, participant and enrollment site, see Methods for more details.

B- B Cell to non-granulocyte frequency: B Cell to non-granulocyte frequency values collected during the first 28 days of hospital admission by four PRO clusters, minimal deficit (MIN, n= 491), physical predominant (PHY, n = 111), deficit, mental/cognitive predominant (COG, n = 126) and deficit, multidomain (MLT, n = 93). The points represent the individual frequency values. The solid black trend line for each PRO cluster represents the fit of a generalized additive model (GAM) to capture non-linear trends in the data. The grey lines represent individual fitted lines for each participant. The B Cell to non-granulocyte frequency was not significantly associated with the PRO clusters (shape adj. p = 0.6686, average adj. p= 0.1012). Statistical differences were determined from generalized additive mixed effects models adjusting for age, sex, participant and enrollment site, see Methods for more details.

C- Naïve B Cell to non-granulocyte frequency: Naïve B Cell to non-granulocyte frequency values collected during the first 28 days of hospital admission by four PRO clusters, minimal deficit (MIN, n= 491), physical predominant (PHY, n = 111), deficit, mental/cognitive predominant (COG, n = 126) and deficit, multidomain (MLT, n = 93). The points represent the individual frequency values. The solid black trend line for each PRO cluster represents the fit of a generalized additive model (GAM) to capture non-linear trends in the data. The grey lines represent individual fitted lines for each participant. The Naïve B Cell to non-granulocyte frequency was not significantly associated with the PRO clusters (shape adj. p = 0.909, average adj. p= 0.4229). Statistical differences were determined from generalized additive mixed effects models adjusting for age, sex, participant and enrollment site, see Methods for more details.

The four PRO clusters are the following in grey: minimal deficit (MIN), in blue: deficit, physical predominant (PHY), in yellow: deficit, mental/cognitive predominant (COG) and in red: deficit, multidomain (MLT). The lines and asterisks on top of the figure denote pairwise statistical significance, * $p < 0.05$, ** $p < 0.01$, *** $p < 0.001$ and

NS denote not significant. P-values were adjusted using the Benjamini-Hochberg method to account for multiple comparisons.

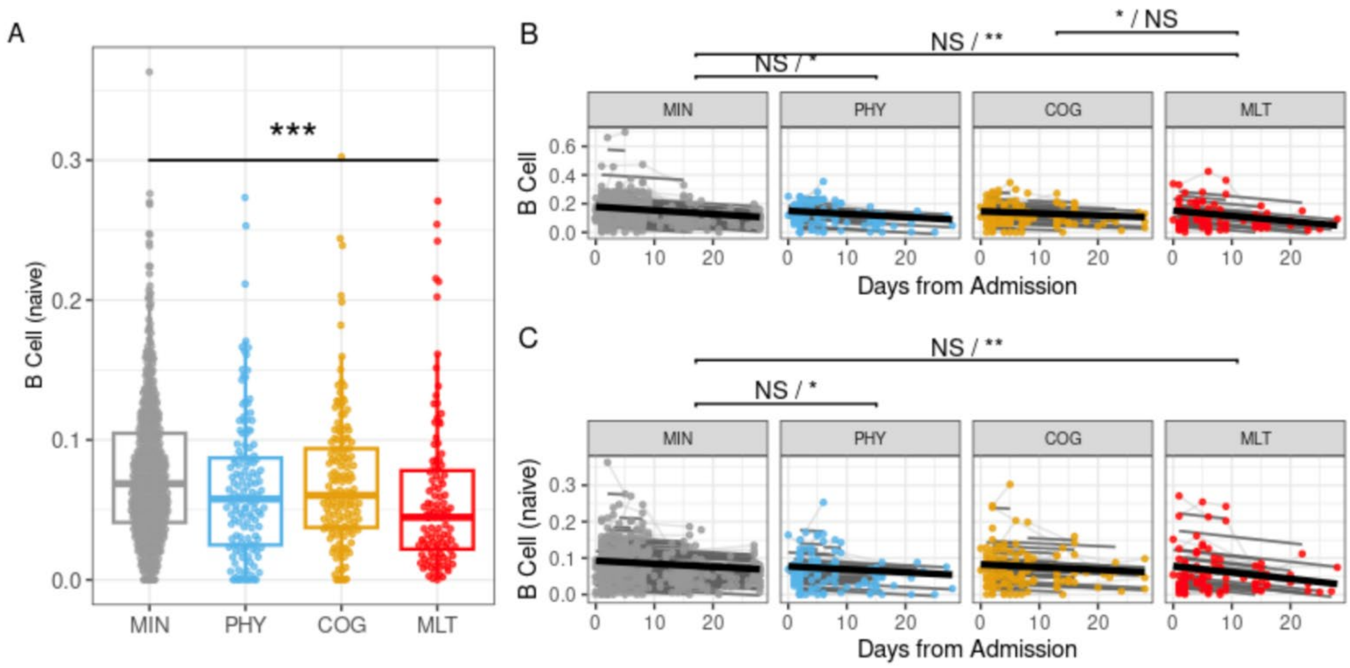

Figure 6S:

A- Ratio of viral load (N1) to B cells frequency: Shown are scaled ratios of viral load (N1) to B cells frequency from samples collected during the first 28 days of hospital admission by four PRO clusters, minimal deficit (MIN, n= 419), physical predominant (PHY, n = 96), deficit, mental/cognitive predominant (COG, n = 98) and deficit, multidomain (MLT, n = 66). Shown are median values (horizontal lines), interquartile ranges (boxes), and 1.5 IQR (whiskers), as well as all individual points. The ratio of viral load (N1) to B cells frequency was also significantly (adj. p= 0.011) associated with the PRO clusters. Statistical differences were determined from generalized linear mixed effects models adjusting for age, sex, participant and enrollment site, see Methods for more details.

B- Ratio of viral load (N1) to B cells frequency over time: Shown are the trends of the scaled ratio of viral load (N1) values divided by B cells frequency for time points collected during the first 28 days of hospital admission by four PRO clusters, minimal deficit (MIN, n= 388), physical predominant (PHY, n = 92), deficit, mental/cognitive predominant (COG, n = 90) and deficit, multidomain (MLT, n = 64). The ratio of viral load (N1) to B cells frequency was also significantly (shape adj. p= 0.93, average adj. p= 0.0086) associated with the PRO clusters. Statistical differences were determined from generalized additive mixed effects models adjusting for age, sex, participant and enrollment site, see Methods for more details.

C- Ratio of viral load (N2) to B cells frequency: Shown are scaled ratios of viral load (N2) to B cells frequency from samples collected during the first 28 days of hospital admission by four PRO clusters, minimal deficit (MIN, n= 419), physical predominant (PHY, n = 96), deficit, mental/cognitive predominant (COG, n = 98) and deficit, multidomain (MLT, n = 66). Shown are median values (horizontal lines), interquartile ranges (boxes), and 1.5 IQR (whiskers), as well as all individual points. The ratio of viral load (N2) to B cells frequency was also significantly (adj. p= 0.011) associated with the PRO clusters. Statistical differences were determined from generalized linear mixed effects models adjusting for age, sex, participant and enrollment site, see Methods for more details.

D- Ratio of viral load (N2) to B cells frequency over time: Shown are the trends of the scaled ratio of viral load (N2) values divided by B cells frequency for time points collected during the first 28 days of hospital admission by four PRO clusters, minimal deficit (MIN, n= 388), physical predominant (PHY, n = 92), deficit, mental/cognitive predominant (COG, n = 90) and deficit, multidomain (MLT, n = 64). The ratio of viral load (N2) to B cells frequency was also significantly (shape adj. p= 0.93, average adj. p= 0.0086) associated with the PRO clusters. Statistical differences were determined from generalized additive mixed effects models adjusting for age, sex, participant and enrollment site, see Methods for more details.

E- Ratio of anti-RBD IgG to B cells frequency: Shown are scaled ratios of anti-RBD IgG to B cells frequency from samples collected during the first 28 days of hospital admission by four PRO clusters, minimal deficit (MIN, n= 621), physical predominant (PHY, n = 134), deficit, mental/cognitive predominant (COG, n = 139) and deficit, multidomain (MLT, n = 100). Shown are median values (horizontal lines), interquartile ranges (boxes), and 1.5 IQR (whiskers), as well as all individual points. The ratio of anti-RBD IgG to B cells frequency was also significantly (adj. p= 0.029) associated with the PRO clusters. Statistical differences were determined from generalized linear mixed effects models adjusting for age, sex, participant and enrollment site, see Methods for more details.

F- Ratio of anti-RBD IgG to B cells frequency over time: Shown are the trends of the scaled ratio of anti-RBD IgG values divided by B cells frequency for time points collected during the first 28 days of hospital admission by four PRO clusters, minimal deficit (MIN, n= 553), physical predominant (PHY, n = 123), deficit, mental/cognitive predominant (COG, n = 124) and deficit, multidomain (MLT, n = 94). The ratio of anti-RBD IgG to B cells frequency was (shape adj. p= 0.28, average adj. p= 0.092) associated with the PRO clusters. Statistical differences were determined from generalized additive mixed effects models adjusting for age, sex, participant and enrollment site, see Methods for more details.

G- Ratio of anti-Spike IgG to B cells frequency: Shown are scaled ratios of anti-Spike IgG to B cells frequency from samples collected during the first 28 days of hospital admission by four PRO clusters, minimal deficit (MIN, n= 621), physical predominant (PHY, n = 134), deficit, mental/cognitive predominant (COG, n = 139) and deficit, multidomain (MLT, n = 100). Shown are median values (horizontal lines), interquartile ranges (boxes), and 1.5 IQR (whiskers), as well as all individual points. The ratio of anti-Spike IgG to B cells frequency was also significantly (adj. p= 0.05) associated with the PRO clusters. Statistical differences were determined from generalized linear mixed effects models adjusting for age, sex, participant and enrollment site, see Methods for more details.

H- Ratio of anti-Spike IgG to B cells frequency over time: Shown are the trends of the scaled ratio of anti-Spike IgG values divided by B cells frequency for time points collected during the first 28 days of hospital admission by four PRO clusters, minimal deficit (MIN, n= 553), physical predominant (PHY, n = 123), deficit, mental/cognitive predominant (COG, n = 124) and deficit, multidomain (MLT, n = 94). The ratio of anti-Spike IgG to B cells frequency was (shape adj. p= 0.22, average adj. p= 0.15) associated with the PRO clusters. Statistical differences were determined from generalized additive mixed effects models adjusting for age, sex, participant and enrollment site, see Methods for more details.

The four PRO clusters are the following in grey: minimal deficit (MIN), in blue: deficit, physical predominant (PHY), in yellow: deficit, mental/cognitive predominant (COG) and in red: deficit, multidomain (MLT). The lines and asterisks on top of the figure denote pairwise statistical significance, * p < 0.05, ** p < 0.01, *** p < 0.001 and NS denote not significant. P-values were adjusted using the Benjamini-Hochberg method to account for multiple comparisons.

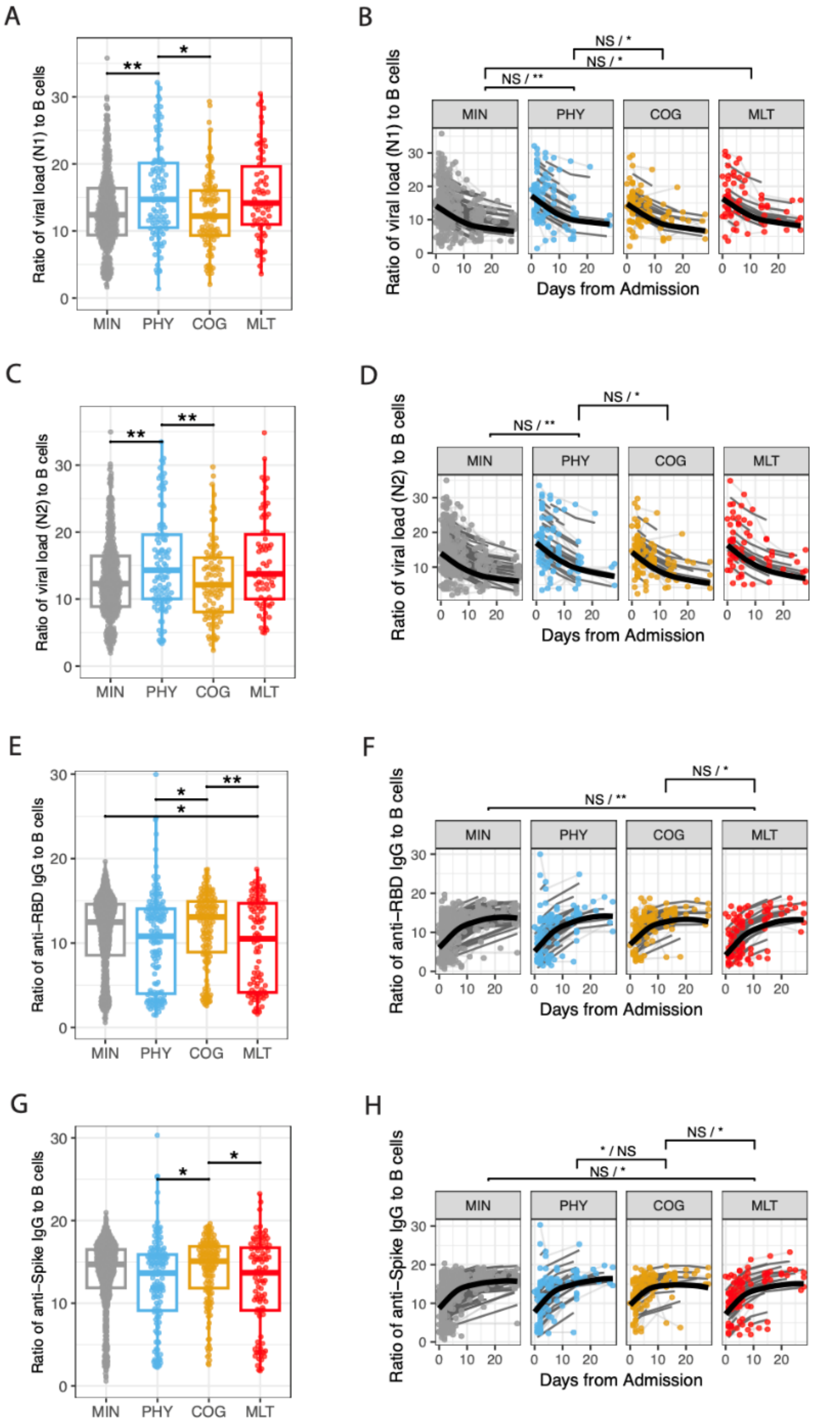

Figure 7S:

A- N1 Ct values: Shown are SARS-CoV-2 N1 gene Ct values (viral loads) measured from samples collected at the earliest time point in the first 28 days of hospital admission by Cases (n = 17) and Controls (n = 52). Shown are median values (horizontal lines), interquartile ranges (boxes), and 1.5 IQR (whiskers), as well as all individual points. Because lower Ct values indicate higher viral loads, the y-axis is reversed.

B- N2 Ct values: Shown are SARS-CoV-2 N2 gene Ct values (viral loads) measured from samples collected at the earliest time point in the first 28 days of hospital admission by Cases (n = 17) and Controls (n = 52). Shown are median values (horizontal lines), interquartile ranges (boxes), and 1.5 IQR (whiskers), as well as all individual points. Because lower Ct values indicate higher viral loads, the y-axis is reversed.

C- Anti-RBD IgG values: Shown are anti-RBD IgG values measured from samples collected at the earliest time point in the first 28 days of hospital admission by Cases (n = 22) and Controls (n = 69). Shown are median values (horizontal lines), interquartile ranges (boxes), and 1.5 IQR (whiskers), as well as all individual points.

D- Anti-Spike IgG values: Shown are anti-Spike IgG values measured from samples collected at the earliest time point in the first 28 days of hospital admission by Cases (n = 22) and Controls (n = 69). Shown are median values (horizontal lines), interquartile ranges (boxes), and 1.5 IQR (whiskers), as well as all individual points.

Cases are participants with positive IFN autoantibodies on admission. Controls are selected in 3:1 ratio (controls to cases) matching by age and sex. The lines and asterisks on top of the figure denote pairwise statistical significance, * $p < 0.05$, ** $p < 0.01$, *** $p < 0.001$ and NS denote not significant. Statistical differences were determined by the Wilcox test. P values are unadjusted and two sided.

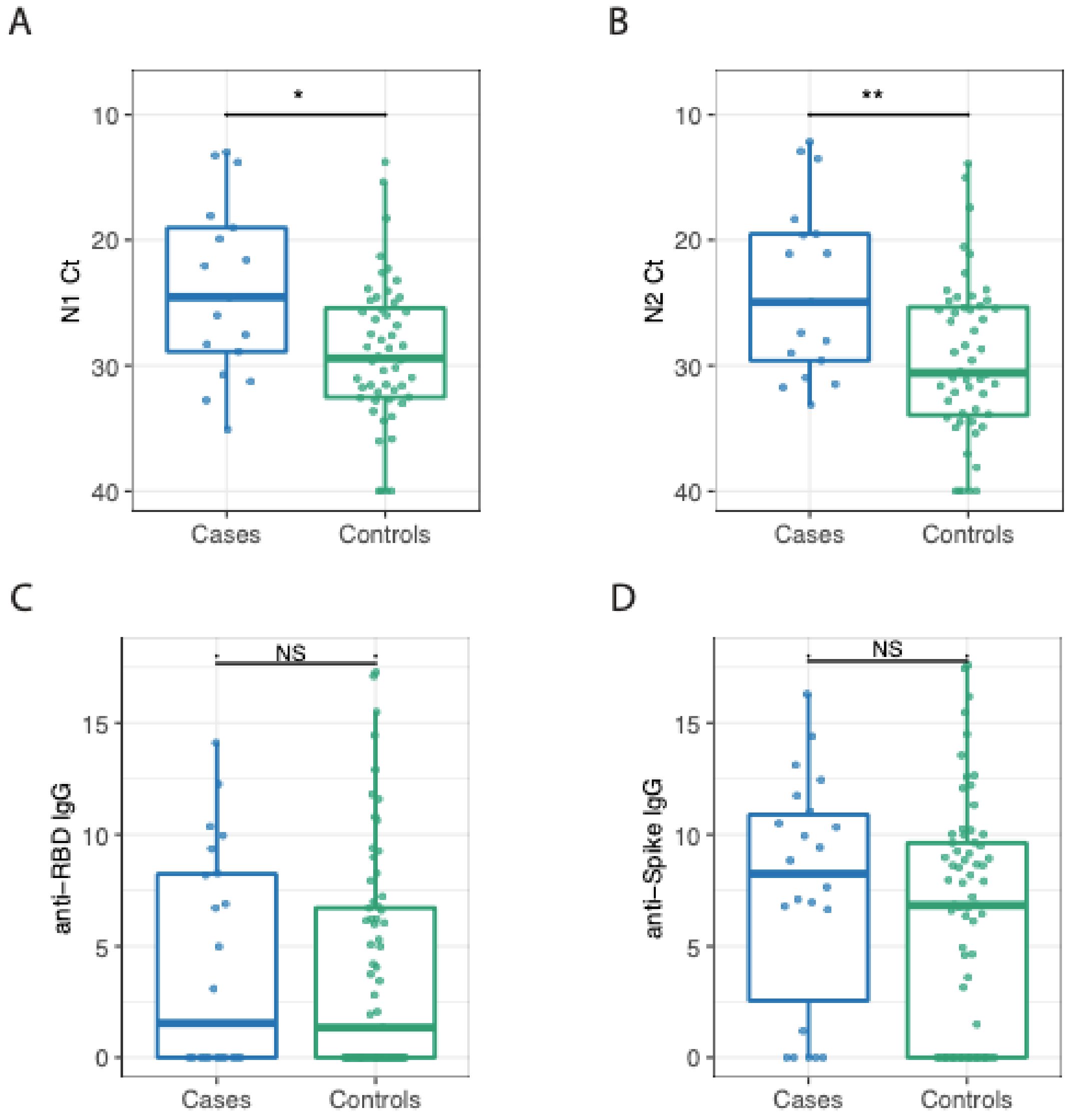

A
B
C
D
N1 Ct
N2 Ct
anti-RBD IgG
anti-Spike IgG
*
**
NS
NS
10
20
30
40
0
5
10
15
Cases
Controls

Figure 8S

FGF21 NPX (Normalized protein expression): FGF21 NPX values collected during the first 28 days of hospital admission by four PRO clusters, minimal deficit (MIN, n= 716), physical predominant (PHY, n = 189), deficit, mental/cognitive predominant (COG, n = 210) and deficit, multidomain (MLT, n = 139). The points represent the individual NPX values. The solid black trend line for each PRO cluster represents the fit of a generalized additive model (GAM) to capture non-linear trends in the data. The grey lines represent individual fitted lines for each participant. The FGF21 NPX was significantly associated with the PRO clusters (shape adj. p = 0.316, average adj. p= 0.0135).

The four PRO clusters are the following in grey: minimal deficit (MIN), in blue: deficit, physical predominant (PHY), in yellow: deficit, mental/cognitive predominant (COG) and in red: deficit, multidomain (MLT). The lines and asterisks on top of the figure denote pairwise statistical significance, * p < 0.05, ** p < 0.01, *** p < 0.001 and NS denote not significant. Statistical differences were determined from generalized additive mixed effects models adjusting for age, sex, participant and enrollment site. P-values were adjusted using the Benjamini-Hochberg method to account for multiple comparisons. See Methods for more details.

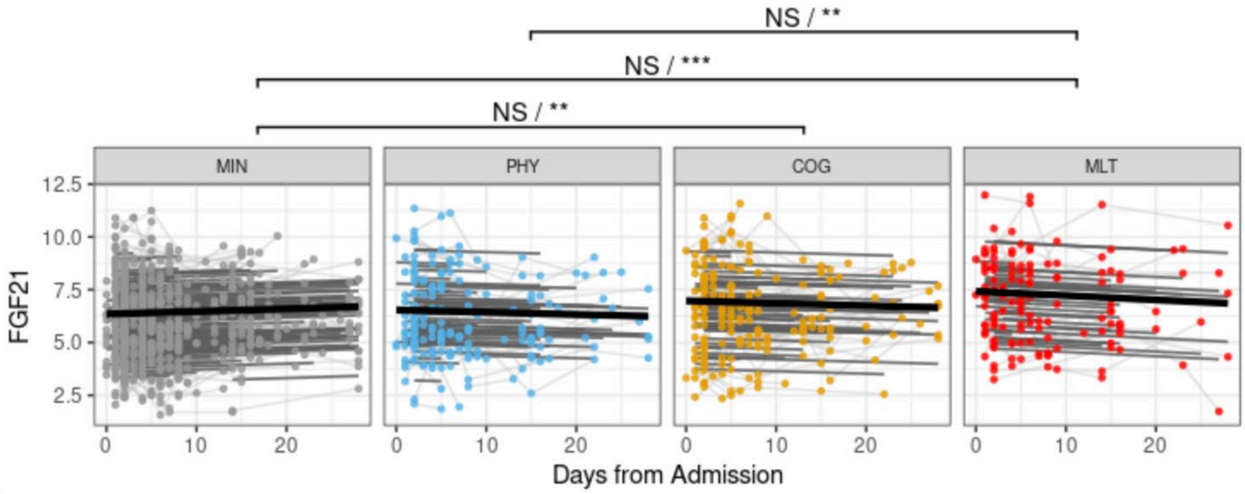

Figure 9S:

A – Global metabolomics WGCNA module 3 (Methylhistidine Metabolism): Shown are eigenvalues for the global metabolomics methylhistidine metabolism module, comprised of 35 metabolites identified from samples taken during the first 28 days of hospitalization by four PRO clusters, minimal deficit (MIN, n= 811), physical predominant (PHY, n = 215), deficit, mental/cognitive predominant (COG, n = 230) and deficit, multidomain (MLT, n = 147). Shown are median values (horizontal lines), interquartile ranges (boxes), and 1.5 IQR (whiskers), as well as all individual points. The repeated measurements model indicates that module 3 is not significantly associated with the PRO clusters (adj. p = 0.509). However, pairwise comparisons between PRO clusters are shown, with significant differences in shape between the minimal deficit (MIN) and physical predominant (PHY) clusters (shape adj. p = 0.0488), as well as the minimal deficit (MIN) and multidomain (MLT) clusters (shape adj. p = 0.0376). Statistical differences were determined from generalized linear mixed effects models adjusting for age, sex, participant and enrollment site, see Methods for more details.

B – Global metabolomics WGCNA module 18 (Acyl-carnitine Metabolism): Shown are eigenvalues for the global metabolomics acyl carnitine metabolism module, comprised of 10 metabolites identified from samples taken during the first 28 days of hospitalization by four PRO clusters, minimal deficit (MIN, n= 811), physical predominant (PHY, n = 215), deficit, mental/cognitive predominant (COG, n = 230) and deficit, multidomain (MLT, n = 147). Shown are median values (horizontal lines), interquartile ranges (boxes), and 1.5 IQR (whiskers), as well as all individual points. The repeated measurements model indicates that module 18 is not significantly associated with the PRO clusters (adj. p = 0.855). Pairwise comparisons between PRO clusters are shown, with significant differences in shape between the minimal deficit (MIN) and physical predominant (PHY) clusters (shape adj. p = 0.0545). Statistical differences were determined from generalized linear mixed effects models adjusting for age, sex, participant and enrollment site, see Methods for more details.

C – Global metabolomics WGCNA module 3 (Methylhistidine Metabolism): Shown are solid black generalized additive model (GAM) trend lines for each PRO cluster (minimal deficit (MIN, n= 894), physical predominant (PHY, n = 237), deficit, mental/cognitive predominant (COG, n = 243) and deficit, multidomain (MLT, n = 159)) to capture non-linear trends in eigenvalues for samples taken during the first 28 days of hospitalization. Points represent individual values and grey lines represent individual fitted lines for each participant. (shape adj.p = 0.003; average adj.p = 0.690). Statistical differences were determined from generalized additive mixed effects models adjusting for age, sex, participant and enrollment site, see Methods for more details.

D - Global metabolomics WGCNA module 18 (Acyl-carnitine Metabolism): Shown are solid black generalized additive model (GAM) trend lines for each PRO cluster (minimal deficit (MIN, n= 894), physical predominant (PHY, n = 237), deficit, mental/cognitive predominant (COG, n = 243) and deficit, multidomain (MLT, n = 159)) to capture non-linear trends in eigenvalues for samples taken during the first 28 days of hospitalization. Points represent individual values and grey lines represent individual fitted lines for each participant. (shape adj.p = 0.015; average adj.p = 0.931). Statistical differences were determined from generalized additive mixed effects models adjusting for age, sex, participant and enrollment site, see Methods for more details.

The four PRO clusters are the following in grey: minimal deficit (MIN), in blue: deficit, physical predominant (PHY), in yellow: deficit, mental/cognitive predominant (COG) and in red: deficit, multidomain (MLT). The lines and asterisks on top of the figure denote pairwise statistical significance, * $p < 0.05$, ** $p < 0.01$, *** $p < 0.001$ and NS denote not significant. P-values were adjusted using the Benjamini-Hochberg method to account for multiple comparisons.

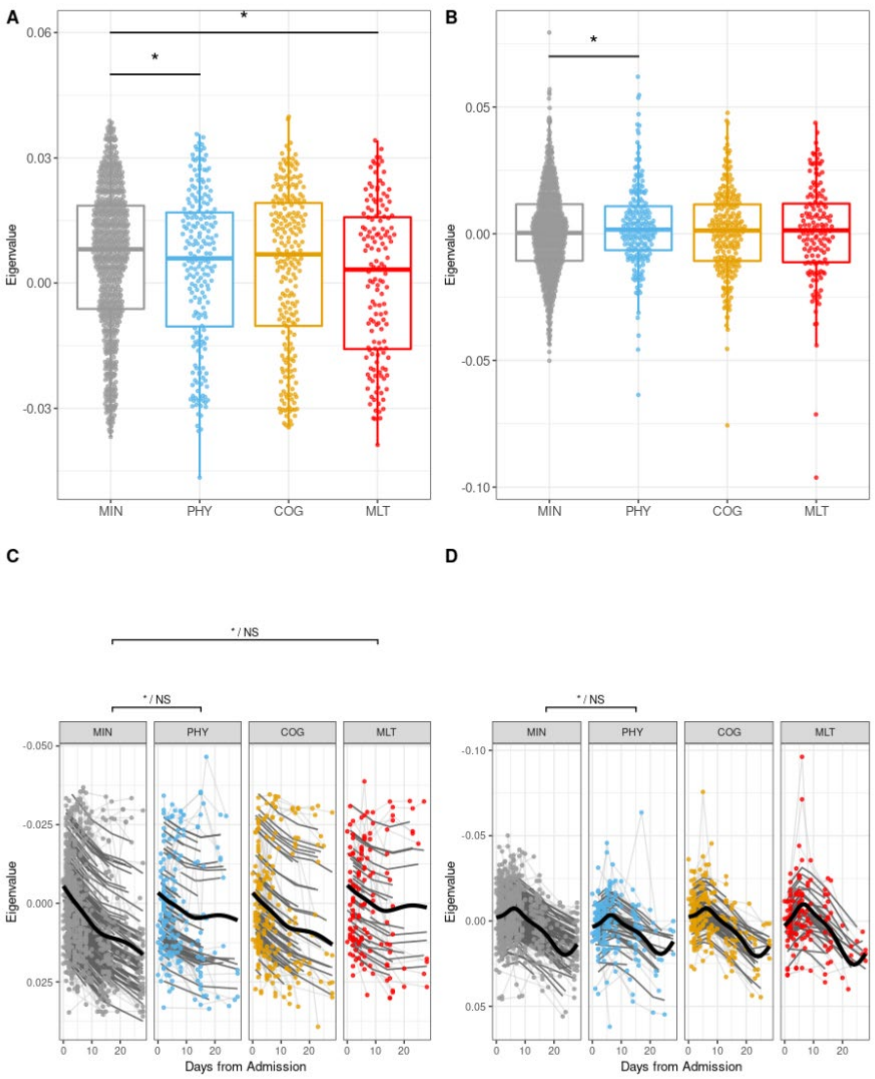
A
B
C
D
Eigenvalue
MIN
PHY
COG
MLT
*
* / NS
Days from Admission

APPENDIX: RESOURCES

REAGENT or RESOURCE	SOURCE	IDENTIFIER
Antibodies		
Maxpar® Direct™ Immune Profiling Assay (MDIPA) Kit	Fluidigm	Cat#201325
CD8a-146Nd	Fluidigm	Cat#3146001B; RRID:AB_2687641
Granzyme B Antibody, anti-human/mouse/rat, REAfinity	Miltenyi	Cat#130-116-486
Goat Anti-Human IgA-UNLB	Southern Biotech	Cat#2050-01
Purified anti-human IgM Antibody	Biolegend	Cat#314502
Mouse Anti-Human IgG1 Fc-UNLB	Souther Biotech	Cat#9054-01
Purified anti-mouse/human CD11b Antibody	Biolegend	Cat#101202
Purified anti-human/mouse/rat CD278 (ICOS) Antibody	Biolegend	Cat#313502
Purified anti-human CD39 Antibody	Biolegend	Cat#328202
Purified anti-human CD169 (Sialoadhesin, Siglec-1) Antibody	Biolegend	Cat#346002
Purified anti-human CD64 (Maxpar® Ready) Antibody	Biolegend	Cat#305029
Purified anti-human CD71 Antibody	Biolegend	Cat#334102
Anti-Human CD279/PD-1 (EH12.2H7)-175Lu	Fluidigm	Cat#3175008B
Anti-Human CD61 (VI-PL2)-209Bi	Fluidigm	Cat#3209001B
Anti-Human CD3 (UCHT1)-141Pr antibody	Fluidigm	Cat#3141019B
Anti-Human HLA-DR (L243)-143Nd antibody	Fluidigm	Cat#3143013B
Anti-Human CD69 (FN50)-144Nd antibody	Fluidigm	Cat#3144018B
Anti-Human CD4 (RPA-T4)-145Nd antibody	Fluidigm	Cat#3145001B
Anti-Human CD8a (RPA-T8)-146Nd antibody	Fluidigm	Cat#3146001B
Anti-Human CD20 (2H7)-147Sm antibody	Fluidigm	Cat#3147001B
Anti-Human CD127 (A019D5)-149Sm antibody	Fluidigm	Cat#3149011B
Anti-Human MIP-1β (D21-1351)-150Nd antibody	Fluidigm	Cat#3150004B
Anti-Human CD123 (6H6)-151Eu antibody	Fluidigm	Cat#3151001B

Anti-Human TNFα (Mab11)-152Sm antibody	Fluidigm	Cat#3152002B
Anti-Human CD62L (DREG-56)-153Eu antibody	Fluidigm	Cat#3153004B
Anti-Human CD45 (HI30)-154Sm antibody	Fluidigm	Cat#3154001B
Anti-Human IL-6 (MQ2-13A5)-156Gd antibody	Fluidigm	Cat#3156011B
Anti-Human IFN-γ (B27)-158Gd antibody	Fluidigm	Cat#3158017B
Anti-Human CD11c (Bu15)-159Tb antibody	Fluidigm	Cat#3159001B
Anti-Human CD14 (M5E2)-160Gd antibody	Fluidigm	Cat#3160001B
Anti-Human CD80/B7.1 (2D10.4)-161Dy antibody	Fluidigm	Cat#3161023B
Anti-Human CD66b (80H3)-162Dy antibody	Fluidigm	Cat#3162023B
Anti-Human CD56 (NCAM16.2)-163Dy antibody	Fluidigm	Cat#3163007B
Anti-Human CD15 (W6D3)-164Dy antibody	Fluidigm	Cat#3164001B
Anti-Human CD61 (VI-PL2)-165Ho antibody	Fluidigm	Cat#3165010B
Anti-Human CD11b (ICRF44)-167Er antibody	Fluidigm	Cat#3167011B
Anti-Human CD206 (15-2)-168Er antibody	Fluidigm	Cat#3168008B
Anti-Human CD54 (HA58)-170Er antibody	Fluidigm	Cat#3170014B
Anti-Human CD68 (Y1/82A)-171Yb antibody	Fluidigm	Cat#3171011B
Anti-Human CD16 (3G8)-209Bi antibody	Fluidigm	Cat#3209002B
Anti- CoV Nucleocapsid protein (6H3) antibody	Abcam	Cat#ab273434
Anti-Human Eotaxin (43915) antibody	R&D	Cat#MAB3201
Anti-Human ACE-2 (535919) antibody	NOVUS	Cat#MAB9332-100
Anti-Human Cytokeratin (C-11) antibody	Biolegend	Cat#628602
Anti- CoV Spike protein (1A9) antibody	GeneTex	Cat#GTX632604
Anti-Human EPX (MM82.2.1) antibody	MAYO CLINIC	https://www.mayoclinic.org
Anti-Human IL-8 (E8N1) antibody	Biolegend	Cat#511402
Anti-Human IL-1β (H1b-27) antibody	Biolegend	Cat#511602
Anti-Human IFN-β (IFNb/A1) antibody	Biolegend	Cat#514002
Anti-Human Siglec-8 (837535) antibody	R&D	Cat#MAB7975

Anti-human IgG (Fc specific)-Peroxidase antibody produced in goat	Sigma-Aldrich	Cat#A0170; RRID: AB_257868
Goat anti-human IgM-HRP	SouthernBiotech	Cat#2020-05; RRID: AB_2795603
Anti-human IgA (α-chain specific)-Peroxidase antibody produced in goat	Sigma-Aldrich	Cat#A0295; RRID: AB_257876
Anti-Glial Fibrillary Associated Protein	Agilent	Cat#Z033429-2
Anti-human IgG (PE)	ThermoScientific	Cat#12-4998-8
Anti-human pSTAT1 (AF647)	BD	Cat#612597
Anti-human CD14 (FITC)	BD	Cat#555397
Bacterial and virus strains		
BLT5403, T7 Select Kit	Novagen	Cat#70550-3
T7 Bacteriophage, T7 Select Kit	Novagen	Cat#70550-3
Biological samples		
Plasma samples from IMPACC cohort	Multiple clinical sites	N/A
Whole blood from hospitalized COVID19 patients-collected in EDTA tubes	Multiple clinical sites	N/A
Veri-Cells™ Heavy Metal (Ta) PBMC	Biolegend	Cat#427203
Serum samples from IMPACC cohort	Multiple clinical sites	N/A
Stimulated Plasma from Healthy Controls	Stanford University	N/A
Plasma from Healthy Controls	Stanford University	N/A
Serum from Healthy Controls	Stanford University	N/A
Chemicals, peptides, and recombinant proteins		
DNA/RNA Shield Collection Tube w/ Swab - DX	Zymo Research	Cat#R1107-E
Quick-DNA/RNA MagBead	Zymo Research	Cat#R2131
Stranded Total RNA Prep, Ligation with Ribo-Zero Plus	Illumina	Cat#20040529
HS NGS Fragment Kit	Agilent	Cat#DNF-474-0500
K-562 Total RNA	Thermo Fisher	Cat#AM7832
qScript XLT 1-Step RT-qPCR ToughMix	Quantabio	Cat#95133-02K

2-propanolol (LC-MS)	MilliporeSigma	Cat#1027814000
Acetonitrile (LC-MS)	MilliporeSigma	Cat# 1000294000
Water, Baker Analyzed LC/MS Reagent Grade	J.T. Baker	Cat#9831-02
Ammonium Formate (LC-MS)	J.T. Baker	Cat#M530-08
Perfluoropentanoic acid	Sigma	Cat#396575
Ammonium Bicarbonate	Fisher	Cat#A643
Ammonium Hydroxide	Sigma	Cat#338818
Cell-ID™ 20-Plex Pd Barcoding Kit	Fluidigm	Cat#201060
Saponin	Sigma	Cat#47036
Human TruStain FcX™ (Fc Receptor Blocking Solution)	Biolegend	Cat#422302; RRID:AB_2818986
Heparin sodium salt	Sigma	Cat#H3393
SmartTube PROT1 stabilizer PROT1-250ML	SmartTube	Fisher Cat# 501351692
SmartTube ThawLyse - THAWLYSE1	SmartTube	Fisher Cat# 501351696
Paraformaldehyde (PFA), 16% w/v aqueous, methanol-free	Alfa Aesar	Fisher Cat# AA433689L
Fetal bovine serum, characterized, heat-inactivated	HyClone	Fisher Cat#SH30396.03
Dimethyl sulfoxide	Fisher	Cat#BP231-100
Maxpar MCP9 Antibody Labeling Kit, 111Cd	Fluidigm	Cat#201111A
Maxpar MCP9 Antibody Labeling Kit, 112Cd	Fluidigm	Cat#201112A
Maxpar MCP9 Antibody Labeling Kit, 114Cd	Fluidigm	Cat#201114A
Maxpar MCP9 Antibody Labeling Kit, 116Cd	Fluidigm	Cat#201116A
Maxpar® X8 Antibody Labeling Kit, 142Nd	Fluidigm	Cat#201142B
Maxpar® X8 Antibody Labeling Kit, 159Tb	Fluidigm	Cat#201159B
Maxpar® X8 Antibody Labeling Kit, 162Dy	Fluidigm	Cat#201162B
Maxpar® X8 Antibody Labeling Kit, 165Ho	Fluidigm	Cat#201165B

Maxpar® X8 Antibody Labeling Kit, 169Tm	Fluidigm	Cat#201169B
Maxpar® X8 Antibody Labeling Kit, 142Nd—4 Rxn	Fluidigm	Cat#201142A
Maxpar® X8 Antibody Labeling Kit, 148Nd—4 Rxn	Fluidigm	Cat#201148A
Maxpar® X8 Antibody Labeling Kit, 155Gd—4 Rxn	Fluidigm	Cat#201155A
Maxpar® X8 Antibody Labeling Kit, 166Er—4 Rxn	Fluidigm	Cat#201166A
Maxpar® X8 Antibody Labeling Kit, 169Tm—4 Rxn	Fluidigm	Cat#201169A
Maxpar® X8 Antibody Labeling Kit, 172Er—4 Rxn	Fluidigm	Cat#201172A
Maxpar® X8 Antibody Labeling Kit, 173Yb—4 Rxn	Fluidigm	Cat#201173A
Maxpar® X8 Antibody Labeling Kit, 174Yb—4 Rxn	Fluidigm	Cat#201174A
Maxpar® X8 Antibody Labeling Kit, 175Lu—4 Rxn	Fluidigm	Cat#201175A
Maxpar® X8 Antibody Labeling Kit, 176Yb—4 Rxn	Fluidigm	Cat#201176A
Cell-ID™ Cisplatin	Fluidigm	Cat#201064
Cell-ID™ Intercalator	Fluidigm	Cat#201192A
Cell-ID™ 20-Plex Pd Barcoding Kit	Fluidigm	Cat#201060
Maxpar® Water—500 mL	Fluidigm	Cat#201069
Maxpar® Cell Staining Buffer	Fluidigm	Cat#201068
Maxpar® PBS	Fluidigm	Cat#201058
EQ Four Element Calibration Beads	Fluidigm	Cat#201078
Bond-Breaker TCEP Solution, Neutral pH	Thermo Fisher	Cat#77720
PFA	EMC	50-980-487
Osmium tetroxide	ACROS ORGANICS	319010050
Recombinant SARS-CoV-2 receptor binding domain (RBD)	Krammer Laboratory at the Icahn School of Medicine at Mount Sinai	https://labs.icahn.mssm.edu/krammerlab/reagents/
Recombinant SARS-CoV-2 spike protein (S)	Krammer Laboratory at the Icahn School of Medicine at Mount Sinai	https://labs.icahn.mssm.edu/krammerlab/reagents/

SIGMAFAST™ OPD (o-Phenylenediamine dihydrochloride)	Sigma-Aldrich	Cat#P9187
3-molar hydrochloric acid	Thermo Fisher Scientific	Cat#S25856
Tween-20	Fisher Bioreagents	Cat#BP337-100
Non-fat dry milk Omniblok	AmericanBio	Cat#AB10109-01000
Bovine Serum Albumin Fraction V	Roche	Cat#10735078001
Protein A conjugated magnetic beads	Invitrogen	Cat#10008D
Protein G conjugated magnetic beads	Invitrogen	Cat#10009D
T4 ligase	New England Biolabs	Cat#M0202S
Phusion DNA Polymerase	New England Biolabs	Cat# M0530L
Urea	Sigma-Aldrich	
Ammonium Bicarbonate	Sigma-Aldrich	09830-1KG
Iodoacetamide	Sigma-Aldrich	I1149-25G
Dithiothreitol	Sigma-Aldrich	D9779-10G
LC/MS grade Formic Acid	Thermo Scientific	A117-50
Perchloric Acid	Sigma-Aldrich	311421-50ML
1-Propanol	Sigma-Aldrich	34871-1L
Sera-Mag Speed Beads 65	Sigma-Aldrich	65152105050250
Sera-Mag Speed Beads 45	Sigma-Aldrich	45152105050250
HPLC grade Water	Fisher chemical	W5-4
LC/MS grade Water	Fisher chemical	W6-1
LC/MS grade Acetonitrile	Fisher chemical	A955-1
HPLC grade Methanol	Fisher chemical	A452-4
LC/MS grade Methanol	Fisher chemical	A456-4
LC/MS grade Isopropanol	Fisher chemical	A461-1
Sequence grade Porcine Trypsin	Promega	V5117
K562 Cell Line Tryptic Peptide Mixture Standard 100 μg	Promega	V6951

Trifluoroacetic acid	Sigma-Aldrich	T6508-100ML
Ambion Nuclease-Free Water	Invitrogen	Cat#AM9937
Recombinant human IFNa	R&D	Cat#11101-2
Recombinant human IFNb	Peprotech	Cat#300-02BC
Recombinant human IFNw	Peprotech	Cat#300-02J
Sulfo-NHS	ThermoScientific	Cat#A39269
EDC	ThermoScientific	Cat#77149
Critical commercial assays		
Olink Target 96 Inflammation Reagent Kit	Olink Proteomics	Cat#95302, Lot#B02101
Experimental models: Cell lines		
Expi293F cells	Thermo Fisher	Cat#A14528
Experimental models: Organisms/strains		
Oligonucleotides		
2019-nCOV_N1-F GAC CCC AAA ATC AGC GAA AT	Integrated DNA technologies	Cat#10006713
2019-nCOV_N1-R TCT GGT TAC TGC CAG TTG AAT CTG	Integrated DNA technologies	Cat#10006713
2019-nCOV_N1-P ACC CCG CAT TAC GTT TGG TGG ACC	Integrated DNA technologies	Cat#10006713
2019-nCOV_N2-F TTA CAA ACA TTG GCC GCA AA	Integrated DNA technologies	Cat#10006713
2019-nCOV_N2-R GCG CGA CAT TCC GAA GAA	Integrated DNA technologies	Cat#10006713
2019-nCOV_N2-P ACA ATT TGC CCC CAG CGC TTC AG	Integrated DNA technologies	Cat#10006713
RP-F AGA TTT GGA CCT GCG AGC G	Integrated DNA technologies	Cat#10006713
RP-R GAG CGG CTG TCT CCA CAA GT	Integrated DNA technologies	Cat#10006713
RP-P TTC TGA CCT GAA GGC TCT GCG CG	Integrated DNA technologies	Cat#10006713

Recombinant DNA		
Vector pCAGGS Containing the SARS-Related Coronavirus 2, Wuhan-Hu-1 Spike Glycoprotein Gene (soluble, stabilized)	BEI Resources	Cat#NR-52394
Vector pCAGGS Containing the SARS-Related Coronavirus 2, Wuhan-Hu-1 Spike Glycoprotein Receptor Binding Domain (RBD)	BEI Resources	Cat#NR-52309
Human Coronavirus Synthetic DNA	Twist Bioscience	https://www.twistbioscience.com
Software		
Metabolon Laboratory Information Management System (LIMS)	Metabolon	Metabolon
MassFragment Application Manager	Waters	Waters MassLynx v.4.1 Waters Corp Milford, USA
Fluidigm software-acquisition, normalization, concatenation v7.0.8493	Fluidigm	https://www.fluidigm.com/products-services/software
Cytobank	Beckman Coulter	https://premium.cytobank.org
R v4.0.2	The Comprehensive R Archive Network	https://cran.r-project.org/
Fluidigm Real-Time PCR Analysis v4.7.1	Fluidigm	https://www.fluidigm.com/products-services/software
Olink NPX Manager v3.3.2.434	Olink Proteomics	https://www.olink.com/products-services/data-analysis-products/npx-manager/
Other		
Turbovap Evaporator	Biotage	Zymark TurboVap Cat#Z-TLVE
Waters Acquity UPLC	Waters	Waters Acquity
BEH C18 columns	Waters	Waters Acquity 2.1 x100 mm, 1.7 um columns

Q-Exactive with Orbitrap mass analyzer	Thermo Scientific	Cat#IQLAAEGAAPFALGMBDK
HILIC columns	Waters UPLC	Waters UPLC BEH Amide 2.1 x 150 mm, 1.7 um
Hamilton MicroLab Star Liquid Handling Robotic System	Hamilton Company	https://www.hamiltoncompany.com/automated-liquid-handling/platforms/microlab-star
Geno/Grinder 2000	SPEX Sample Prep	Geno/Grinder 2000
0.45µm filter plates	Arctic White	AWFP-F20022
1000 ul Pipette Tips	Opentrons	991-00005
300 ul Pipette Tips	Opentrons	991-00008
20 ul Pipette Tips	Opentrons	999-00014
10 ul Pipette Tips	Opentrons	999-00014
20 ul Pipette Tips	Axygen	T-20-R-S
200 ul Pipette Tips	Axygen	T-200-C-L-R-S
Sealing tape 96-well Plates	4titude	4ti-0581
25ml Reservoir	Argos	B3125-100
4-well Reservoir	Axygen	RES-MW4-HP
12-well Reservoir	Axygen	RES16MC-12-N
0.5 ml 96-well Plates	VWR	76210-520
0.8 ml 96-well Plates	VWR	76210-524

SURVEYS ADMINISTERED

Global Health

Please respond to each question or statement by marking one box per row.

		Excellent	Very good	Good	Fair	Poor
Global01	In general, would you say your health is:	☐ 5	☐ 4	☐ 3	☐ 2	☐ 1
Global02	In general, would you say your quality of life is:	☐ 5	☐ 4	☐ 3	☐ 2	☐ 1
Global03	In general, how would you rate your physical health?	☐ 5	☐ 4	☐ 3	☐ 2	☐ 1
Global04	In general, how would you rate your mental health, including your mood and your ability to think?	☐ 5	☐ 4	☐ 3	☐ 2	☐ 1
Global05	In general, how would you rate your satisfaction with your social activities and relationships?	☐ 5	☐ 4	☐ 3	☐ 2	☐ 1
Global09r	In general, please rate how well you carry out your usual social activities and roles. (This includes activities at home, at work and in your community, and responsibilities as a parent, child, spouse, employee, friend, etc.)......	☐ 5	☐ 4	☐ 3	☐ 2	☐ 1

		Completely	Mostly	Moderately	A little	Not at all
Global06	To what extent are you able to carry out your everyday physical activities such as walking, climbing stairs, carrying groceries, or moving a chair?	☐ 5	☐ 4	☐ 3	☐ 2	☐ 1

In the past 7 days…

		Never	Rarely	Sometimes	Often	Always
Global10r	How often have you been bothered by emotional problems such as feeling anxious, depressed or irritable?..	☐ 5	☐ 4	☐ 3	☐ 2	☐ 1

		None	Mild	Moderate	Severe	Very severe
Global08r	How would you rate your fatigue on average?	☐ 5	☐ 4	☐ 3	☐ 2	☐ 1

Global07r	How would you rate your pain on average?	☐ 0 **No pain**	☐ 1	☐ 2	☐ 3	☐ 4	☐ 5	☐ 6	☐ 7	☐ 8	☐ 9	☐ 10 **Vorst pain imaginable**

MYOWNMED SYSTEM GENERATED SURVEYS:

DAY-14 Survey

Did you complete this survey by yourself?

--SELECT--

Think about what you could do physically and mentally before your COVID hospitalization. On a scale of 1 to 100, with 100 being all the way back to what you could do before COVID, how close to being back are you?

For the next questions, please select the answer that best describes your health today.

Mobility

--SELECT--

Self-Care

--SELECT--

Usual Activities (e.g. work, study, housework, family or leisure activities)

--SELECT--

Pain / Discomfort

--SELECT--

Anxiety / Depression

--SELECT--

Have you have any symptoms over the past 7 days? (For example: loss of taste, loss of smell, red/watery eyes, shortness of breath, cough, headache, body aches, sore throat, severe fatigue, fever, vomiting/diarrhea, or chills)

--SELECT--

Enter the date this survey was completed. ***Please do not enter a future date.***

month/day/year

DAY-28 Survey

Did you complete this survey by yourself?

--SELECT--

Think about what you could do physically and mentally before your COVID hospitalization. On a scale of 1 to 100, with 100 being all the way back to what you could do before COVID, how close to being back are you?

For the next questions, please select the answer that best describes your health over the past 7 days.

Mobility

--SELECT--

Self-Care

--SELECT--

Usual Activities (e.g. work, study, housework, family or leisure activities)

--SELECT--

Pain / Discomfort

--SELECT--

Anxiety / Depression

--SELECT--

Have you have any symptoms over the past 7 days? (For example: loss of taste, loss of smell, red/watery eyes, shortness of breath, cough, headache, body aches, sore throat, severe fatigue, fever, vomiting/diarrhea, or chills)

--SELECT--

Enter the date this survey was completed. ***Please do not enter a future date.***

month/day/year

MONTH-3 Survey

Did you complete this survey by yourself?

Think about what you could do physically and mentally before your COVIO hospitalization. On a scale of 1 to 100, with 100 being all the way back to what you could do before COVIO, how close to being back are you?

For the next questions, please select the answer that best describes your health over the past 3 months.

Mobility
--SELECT--

Self-Ca re
--SELECT--

Usual Activities (e.g. work, study, housework, family or leisure activities)
--SELECT--

Pain / Discomfort
--SELECT--

Anxiety / Depression
--SELECT--

Have you have any symptoms over the past 7 days? (For example: loss of taste, loss of smell, red/watery eyes, shortness of breath, cough, headache, body aches, sore throat, severe fatigue, fever, vomiting/diarrhea, or chills)
--SELECT--

Did you have an unscheduled doctors visit since your last assessment?
--SELECT--

Did you have any infection requiring antibiotics?
--SELECT--

Were you hospitalized since your last assessment?
--SELECT--

Do you have shortness of breath?
--SELECT--

In general, how would you rate your mental health, including your mood and your ability to think?
--SELECT--

In general, how would you rate your satisfaction with your social activities and relationships?
- SELECT--

Thinking about how your illness has affected you, please rate how true these stat ments were of you BEFORE your illness, and again NOW, since your illness.

I am comfortable with who I am. How true was this before your illness?
--SELECT--

I am comfortable with who I am. How true is this now, since your illness?
--SELECT--

I realize who my real friends are. How true was this before your illness?
--SELECT--

I realize who my real friends are. How true is this now, since your illness?
--SELECT--

l can adjust to things l cannot change. How true was this before your illness?
--SELECT--

l can adjust to things l cannot change. Ho w true is this now, since you r illness?
--SELECT--

My life is meaningful. How true was this before your illness?
--SELECT--

My life is meaningful. How true is this now, since your illness?
--SELECT--

I am a n optimistic person. How true was this before your illness?
--SELECT--

I am an optimistic person. How true is this now, since your illness?

--SELECT--

My relationships are meaningful. How true was this before your illness?

--SELECT--

My relationships are meaningful. How true is this now, since your illness?

--SELECT--

I look at things in a positive way. How true was this before your illness?

--SELECT--

I look at things in a positive way. How true is this now, since your illness?

--SELECT--

I can appreciate each day fully. How true was this before your illness?

--SELECT--

I can appreciate each day fully. How true is this now, since your illness?

--SELECT--

For the next questions, please record the vaccines you may have received since your last visit.

Since your last visit, have you received a COVID-19 vaccine (first shot and/or second shot)?

--SELECT--

Did you receive a 3rd dose or booster COVID-19 vaccine?

--SELECT--

Did you receive a flu vaccine for the 2021/2022 flu season?

--SELECT--

Enter the date this survey was completed. ***Please do not enter a future date.***

month/day/year

6-Month Survey

Did you complete this survey by yourself?

--SELECT--

Think about what you could do physically and mentally before your COVID hospitalization. On a scale of 1 to 100, with 100 being all the way back to what you could do before COVID, how close to being back are you?

For the next questions, please select the answer that best describes your health over the past 3 months.

Mobility

--SELECT--

Self-Care

--SELECT--

Usual Activities (e.g. work, study, housework, family or leisure activities)

--SELECT--

Pain / Discomfort

--SELECT--

Anxiety / Depression

--SELECT--

Have you have any symptoms over the past 7 days? (For example: loss of taste, loss of smell, red/watery eyes, shortness of breath, cough, headache, body aches, sore throat, severe fatigue, fever, vomiting/diarrhea, or chills)

--SELECT--

Did you have an unscheduled doctors visit since your last assessment?

--SELECT--

Did you have any infection requiring antibiotics?

--SELECT--

Were you hospitalized since your last assessment?
--SELECT--

Do you have shortness of breath?
--SELECT--

In general, how would you rate your mental health, including your mood and your ability to think?
--SELECT--

In general, how would you rate your satisfaction with your social activities and relationships?
--SELECT--

Thinking about how your illness has affected you, please rate how true these statements were of you BEFORE your illness, and again NOW, since your illness.

I am comfortable with who I am. How true was this before your illness?
--SELECT--

I am comfortable with who I am. How true is this now, since your illness?
--SELECT--

I realize who my real friends are. How true was this before your illness?
--SELECT--

I realize who my real friends are. How true is this now, since your illness?
--SELECT--

I can adjust to things I cannot change. How true was this before your illness?
--SELECT--

I can adjust to things I cannot change. How true is this now, since your illness?
--SELECT--

My life is meaningful. How true was this before your illness?
--SELECT--

My life is meaningful. How true is this now, since your illness?
--SELECT--

I am an optimistic person. How true was this before your illness?
--SELECT--

I am an optimistic person. How true is this now, since your illness?
--SELECT--

My relationships are meaningful. How true was this before your illness?
--SELECT--

My relationships are meaningful. How true is this now, since your illness?
--SELECT--

I look at things in a positive way. How true was this before your illness?
--SELECT--

I look at things in a positive way. How true is this now, since your illness?
--SELECT--

I can appreciate each day fully. How true was this before your illness?
--SELECT--

I can appreciate each day fully. How true is this now, since your illness?
--SELECT--

For the next questions, please record the vaccines you may have received since your last visit.

Since your last visit, have you received a COVID-19 vaccine (first shot and/or second shot)?
--SELECT--

Did you receive a 3rd dose or booster COVID-19 vaccine?
--SELECT--

Did you receive a flu vaccine for the 2021/2022 flu season?
--SELECT--

Enter the date this survey was completed. ***Please do not enter a future date.***
month/day/year

MONTH-9 Survey

Did you complete this survey by yourself?
-SELECT--

Think about what you could do physically and mentally before your COVIO hospitalization. On a scale of 1 to 100, with 100 being all the way back to what you could do before COVIO, how close to being back are you?

For the next questions, please select the answer that best describes your health over the past 3 months.

Mobility
--SELECT--

Self-Care
-SELECT--

Usual Activities (e.g. work, study, housework, family or leisure activities)
--SELECT--

Pain / Dis comfort
--SELECT--

Anxiety/Depression
-SELECT--

Have you have any symptoms over the past 7 days? (For example: loss of taste, loss of smell, red/watery eyes, shortness of breath, cough, headache, body aches, sore throat, severe fatigue, fever, vomiting/diarrhea, or chills)
-SELECT--

Did you have an unscheduled doctors visit since your last assessment?
--SELECT--

Did you have any infection requiring antibiotics?
-SELECT--

Were you hospitalized since your last assessment?
-SELECT--

- SELECT--

Do you have shortness of breath?

--SELECT--

ln general, how would you rate your mental health, including your mood and your ability to think?

In general, how would you rate your satisfaction with your social activities and relationships?
--SELECT--

Thinking about how your illness has affected you, please rate how true these statements were of you BEFORE your illness, and again NOW, since your illness.

--SELECT--

I am comfortable with who I am. How true was this before your illness?

--SELECT--

I am comfortable with who I am. How true is this now, since your illness?

I realize who my real friends are. How true was this before your illness?
-SELECT--

--SELECT--

I realize who my real friends are. How true is this now, since your illness?

--SELECT--

l can adjust to things l cannot change. How true was this before your illness?

- SELECT--

l can adjust to things l cannot change. How true is this now, since your illness?

--SELECT--

My life is meaningful. How true was this before your illness?

My life is meaningful. How true is this now, since your illness?
-SELECT--

--SELECT--

I am an optimistic person. How true was this before your illness?

I am an optimistic person. How true is this now, since your illness?

--SELECT--

My relationships are meaningful. How true was this before your illness?

--SELECT--

My relationships are meaningful. How true is this now, since your illness?

--SELECT--

I look at things in a positive way. How true was this before your illness?

--SELECT--

I look at things in a positive way. How true is this now, since your illness?

--SELECT--

I can appreciate each day fully. How true was this before your illness?

--SELECT--

I can appreciate each day fully. How true is this now, since your illness?

--SELECT--

For the next questions, please record the vaccines you may have received since your last visit.

Since your last visit, have you received a COVID-19 vaccine (first shot and/or second shot)?

--SELECT--

Did you receive a 3rd dose or booster COVID-19 vaccine?

--SELECT--

Did you receive a flu vaccine for the 2021/2022 flu season?

--SELECT--

Enter the date this survey was completed. ***Please do not enter a future date.***

month/day/year

MONTH-12 Survey

Did you complete this survey by yourself?

--SELECT--

Think about what you could do physically and mentally before your COVID hospitalization. On a scale of 1 to 100, with 100 being all the way back to what you could do before COVID, how close to being back are you?

For the next questions, please select the answer that best describes your health over the past 3 months.

Mobility

--SELECT--

Self-Care

--SELECT--

Usual Activities (e.g. work, study, housework, family or leisure activities)

--SELECT--

Pain / Discomfort

--SELECT--

Anxiety / Depression

--SELECT--

Have you have any symptoms over the past 7 days? (For example: loss of taste, loss of smell, red/watery eyes, shortness of breath, cough, headache, body aches, sore throat, severe fatigue, fever, vomiting/diarrhea, or chills)

--SELECT--

Did you have an unscheduled doctors visit since your last assessment?

--SELECT--

Did you have any infection requiring antibiotics?

--SELECT--

Were you hospitalized since your last assessment?

--SELECT--

Do you have shortness of breath?
-SELECT--

In general, how would you rate your menta l health, including your mood and your ability to think?
--SELECT--

ln general, how would you rate your satisfaction with your social activities and relationships?
--SELECT--

Thinking about how your illness has affected you, please rate how true these statements were of you BEFORE your illness, and again NOW, since your illness.

I am comfortable with who I am. How true was this before your illness?
-SELECT--

I am comfortable with who I am. How true is this now, since your illness?
--SELECT--

I realize who my real friends are. How true was this before your illness?
--SELECT--

I realize who my real friends are. How true is this now, since your illness?
-SELECT--

L can adjust to things l cannot change. How true was this before your illness?
--SELECT--

I can adjust to things l cannot change. How true is this now, since your illness?
-SELECT--

My life is meaningful. How true was this before your illness?
--SELECT--

My life is meaningful. How true is this now, since your illness?
--SELECT--

I am an optimistic person. How true was this before your illness?
--SELECT--

I am an optimistic person. How true is this now, since your illness?
-SELECT--

[illegible]

My relationships are meaningful. How true was this before your illness?
-·SELECT-

My relationships are meaningful. How true is this now, since your illness?
--SELECT-

I look at things in a positive way. How true was this before your illness?
--SELECT-

I look at things in a positive way. Ho w true is this now, since your illness?
--SELECT-

I can appreciate each day fully. How true was this before your illness?
--SELECT--

I can appreciate each day fully. How true it is now, since your illness?
-·SELECT--

For the next questions, please record the vaccines you may have received since your last visit.

Since your last visit, have you received a C0VID-19 vaccine (first shot and/or second shot)?
--SELECT--

Did you receive a 3rd dose or booster C0VID-19 vaccine?
--SELECT--

Did you receive a flu vaccine for the 2021/2022fluseason?
--SELECT--

Enter the date this survey was complete month/day /year

© [illegible]

PROMIS Item Bank v1.0-Psychosocial Illness Impact-

Psychosocial Illness Impact-Positive

Please respond to each question or statement by marking one box per row.

Thinking about how your illness has affected you, please rate how true these statements were of you before your illness, and again now, since your illness.

		Not at all	A little bit	Somewhat	Quite a bit	Very much
II2	I am comfortable with who I am					
II2-B	How true was this before your illness?	☐ 2	☐ 2	☐ 3	☐ 4	☐ 5
II2-A	How true is this now, since your illness?	☐ 2	☐ 2	☐ 3	☐ 4	☐ 5

		Not at all	A little bit	Somewhat	Quite a bit	Very much
II3	I believe I can handle problems					
II3-B	How true was this before your illness?	☐ 2	☐ 2	☐ 3	☐ 4	☐ 5
II3-A	How true is this now, since your illness?	☐ 2	☐ 2	☐ 3	☐ 4	☐ 5

		Not at all	A little bit	Somewhat	Quite a bit	Very much
II4	I believe I am a confident person					
II4-B	How true was this before your illness?	☐ 2	☐ 2	☐ 3	☐ 4	☐ 5
II4-A	How true is this now, since your illness?	☐ 2	☐ 2	☐ 3	☐ 4	☐ 5

		Not at all	A little bit	Somewhat	Quite a bit	Very much
II5	I believe I am a good person					
II5-B	How true was this before your illness?	☐ 2	☐ 2	☐ 3	☐ 4	☐ 5
II5-A	How true is this now, since your illness?	☐ 2	☐ 2	☐ 3	☐ 4	☐ 5

		Not at all	A little bit	Somewhat	Quite a bit	Very much
II6	I appreciate the health of my body					
II6-B	How true was this before your illness?	☐ 2	☐ 2	☐ 3	☐ 4	☐ 5
II6-A	How true is this now, since your illness?	☐ 2	☐ 2	☐ 3	☐ 4	☐ 5

© [illegible]

<u>Thinking about how your illness has affected you</u>, please rate how true these statements were of you <u>before your illness</u>, and again now, <u>since your illness.</u>

		Not at all	A little bit	Somewhat	Quite a bit	Very much
II7	I am an optimistic person					
II7-B	How true was this before your illness?	☐ 2	☐ 2	☐ 3	☐ 4	☐ 5
II7-A	How true is this now, since your illness?	☐ 2	☐ 2	☐ 3	☐ 4	☐ 5

		Not at all	A little bit	Somewhat	Quite a bit	Very much
II8	I can keep going when problems arise					
II8-B	How true was this before your illness?	☐ 2	☐ 2	☐ 3	☐ 4	☐ 5
II8-A	How true is this now, since your illness?	☐ 2	☐ 2	☐ 3	☐ 4	☐ 5

		Not at all	A little bit	Somewhat	Quite a bit	Very much
II9	I can handle most anything					
II9-B	How true was this before your illness?	☐ 2	☐ 2	☐ 3	☐ 4	☐ 5
II9-A	How true is this now, since your illness?	☐ 2	☐ 2	☐ 3	☐ 4	☐ 5

		Not at all	A little bit	Somewhat	Quite a bit	Very much
II10	I believe I am a patient person					
II10-B	How true was this before your illness?	☐ 2	☐ 2	☐ 3	☐ 4	☐ 5
II10-A	How true is this now, since your illness?	☐ 2	☐ 2	☐ 3	☐ 4	☐ 5

		Not at all	A little bit	Somewhat	Quite a bit	Very much
II11	I believe I am an honest person					
II11-B	How true was this before your illness?	☐ 2	☐ 2	☐ 3	☐ 4	☐ 5
II11-A	How true is this now, since your illness?	☐ 2	☐ 2	☐ 3	☐ 4	☐ 5

		Not at all	A little bit	Somewhat	Quite a bit	Very much
II12	I know who I can count on in times of trouble					
II12-B	How true was this before your illness?	☐ 2	☐ 2	☐ 3	☐ 4	☐ 5
II12-A	How true is this now, since your illness?	☐ 2	☐ 2	☐ 3	☐ 4	☐ 5

[illegible]

Thinking about how your illness has affected you, please rate how true these statements were of you before your illness, and again now, since your illness.

		Not at all	A little bit	Somewhat	Quite a bit	Very much
II13	I have compassion for others					
II13-B	How true was this before your illness?	☐ 2	☐ 2	☐ 3	☐ 4	☐ 5
II13-A	How true is this now, since your illness?	☐ 2	☐ 2	☐ 3	☐ 4	☐ 5

		Not at all	A little bit	Somewhat	Quite a bit	Very much
II15	My relationships are meaningful					
II15-B	How true was this before your illness?	☐ 2	☐ 2	☐ 3	☐ 4	☐ 5
II15-A	How true is this now, since your illness?	☐ 2	☐ 2	☐ 3	☐ 4	☐ 5

		Not at all	A little bit	Somewhat	Quite a bit	Very much
II16	I am aware of the love and support available from other people					
II16-B	How true was this before your illness?	☐ 2	☐ 2	☐ 3	☐ 4	☐ 5
II16-A	How true is this now, since your illness?	☐ 2	☐ 2	☐ 3	☐ 4	☐ 5

		Not at all	A little bit	Somewhat	Quite a bit	Very much
II17	I realize who my real friends are					
II17-B	How true was this before your illness?	☐ 2	☐ 2	☐ 3	☐ 4	☐ 5
II17-A	How true is this now, since your illness?	☐ 2	☐ 2	☐ 3	☐ 4	☐ 5

		Not at all	A little bit	Somewhat	Quite a bit	Very much
II18	I am comfortable receiving help from others					
II18-B	How true was this before your illness?	☐ 2	☐ 2	☐ 3	☐ 4	☐ 5
II18-A	How true is this now, since your illness?	☐ 2	☐ 2	☐ 3	☐ 4	☐ 5

© [illegible]

<u>Thinking about how your illness has affected you</u>, please rate how true these statements were of you <u>before your illness</u>, and again now, <u>since your illness.</u>

		Not at all	A little bit	Somewhat	Quite a bit	Very much
II19	I can appreciate people in my life					
II19-B	How true was this before your illness?	☐ 2	☐ 2	☐ 3	☐ 4	☐ 5
II19-A	How true is this now, since your illness?	☐ 2	☐ 2	☐ 3	☐ 4	☐ 5

		Not at all	A little bit	Somewhat	Quite a bit	Very much
II20	I am willing to help others					
II20-B	How true was this before your illness?	☐ 2	☐ 2	☐ 3	☐ 4	☐ 5
II20-A	How true is this now, since your illness?	☐ 2	☐ 2	☐ 3	☐ 4	☐ 5

		Not at all	A little bit	Somewhat	Quite a bit	Very much
II21	I make time for family and friends					
II21-B	How true was this before your illness?	☐ 2	☐ 2	☐ 3	☐ 4	☐ 5
II21-A	How true is this now, since your illness?	☐ 2	☐ 2	☐ 3	☐ 4	☐ 5

		Not at all	A little bit	Somewhat	Quite a bit	Very much
II22	I feel connected to people in my community					
II22-B	How true was this before your illness?	☐ 2	☐ 2	☐ 3	☐ 4	☐ 5
II22-A	How true is this now, since your illness?	☐ 2	☐ 2	☐ 3	☐ 4	☐ 5

		Not at all	A little bit	Somewhat	Quite a bit	Very much
II23	I feel close to people I care about					
II23-B	How true was this before your illness?	☐ 2	☐ 2	☐ 3	☐ 4	☐ 5
II23-A	How true is this now, since your illness?	☐ 2	☐ 2	☐ 3	☐ 4	☐ 5

		Not at all	A little bit	Somewhat	Quite a bit	Very much
II24	I am willing to express my emotions					
II24-B	How true was this before your illness?	☐ 2	☐ 2	☐ 3	☐ 4	☐ 5
II24-A	How true is this now, since your illness?	☐ 2	☐ 2	☐ 3	☐ 4	☐ 5

© [illegible]

Thinking about how your illness has affected you, please rate how true these statements were of you before your illness, and again now, since your illness.

II25	I am able to accept the way things work out					
		**Not at all**	**A little bit**	**Somewhat**	**Quite a bit**	**Very much**
II25-B	How true was this before your illness?	☐ 2	☐ 2	☐ 3	☐ 4	☐ 5
II25-A	How true is this now, since your illness?	☐ 2	☐ 2	☐ 3	☐ 4	☐ 5

II26	I can deal with uncertainty					
		**Not at all**	**A little bit**	**Somewhat**	**Quite a bit**	**Very much**
II26-B	How true was this before your illness?	☐ 2	☐ 2	☐ 3	☐ 4	☐ 5
II26-A	How true is this now, since your illness?	☐ 2	☐ 2	☐ 3	☐ 4	☐ 5

II27	I can adjust to things I cannot change					
		**Not at all**	**A little bit**	**Somewhat**	**Quite a bit**	**Very much**
II27-B	How true was this before your illness?	☐ 2	☐ 2	☐ 3	☐ 4	☐ 5
II27-A	How true is this now, since your illness?	☐ 2	☐ 2	☐ 3	☐ 4	☐ 5

II28	I am able to take things as they come					
		**Not at all**	**A little bit**	**Somewhat**	**Quite a bit**	**Very much**
II28-B	How true was this before your illness?	☐ 2	☐ 2	☐ 3	☐ 4	☐ 5
II28-A	How true is this now, since your illness?	☐ 2	☐ 2	☐ 3	☐ 4	☐ 5

II29	I am able to deal with stress and problems					
		**Not at all**	**A little bit**	**Somewhat**	**Quite a bit**	**Very much**
II29-B	How true was this before your illness?	☐ 2	☐ 2	☐ 3	☐ 4	☐ 5
II29-A	How true is this now, since your illness?	☐ 2	☐ 2	☐ 3	☐ 4	☐ 5

II30	I tend to be accepting of things					
		**Not at all**	**A little bit**	**Somewhat**	**Quite a bit**	**Very much**
II30-B	How true was this before your illness?	☐ 2	☐ 2	☐ 3	☐ 4	☐ 5
II30-A	How true is this now, since your illness?	☐ 2	☐ 2	☐ 3	☐ 4	☐ 5

© [illegible]

<u>Thinking about how your illness has affected you</u>, please rate how true these statements were of you <u>before your</u> <u>illness</u>, and again now, <u>since your illness.</u>

		Not at all	A little bit	Somewhat	Quite a bit	Very much
II31	I take good care of myself					
II31-B	How true was this <u>before your illness</u>?	☐ 2	☐ 2	☐ 3	☐ 4	☐ 5
II31-A	How true is this now, <u>since your illness</u>?	☐ 2	☐ 2	☐ 3	☐ 4	☐ 5

		Not at all	A little bit	Somewhat	Quite a bit	Very much
II32	I look at things in a positive way					
II32-B	How true was this <u>before your illness</u>?	☐ 2	☐ 2	☐ 3	☐ 4	☐ 5
II32-A	How true is this now, <u>since your illness</u>?	☐ 2	☐ 2	☐ 3	☐ 4	☐ 5

		Not at all	A little bit	Somewhat	Quite a bit	Very much
II33	I am able to feel joy					
II33-B	How true was this <u>before your illness</u>?	☐ 2	☐ 2	☐ 3	☐ 4	☐ 5
II33-A	How true is this now, <u>since your illness</u>?	☐ 2	☐ 2	☐ 3	☐ 4	☐ 5

		Not at all	A little bit	Somewhat	Quite a bit	Very much
II34	I am able to enjoy life					
II34-B	How true was this <u>before your illness</u>?	☐ 2	☐ 2	☐ 3	☐ 4	☐ 5
II34-A	How true is this now, <u>since your</u> <u>illness</u>?	☐ 2	☐ 2	☐ 3	☐ 4	☐ 5

		Not at all	A little bit	Somewhat	Quite a bit	Very much
II35	I can appreciate each day fully					
II35-B	How true was this <u>before your illness</u>?	☐ 2	☐ 2	☐ 3	☐ 4	☐ 5
II35-A	How true is this now, <u>since your illness</u>?	☐ 2	☐ 2	☐ 3	☐ 4	☐ 5

[illegible]

Thinking about how your illness has affected you, please rate how true these statements were of you before your illness, and again now, since your illness.

		Not at all	A little bit	Somewhat	Quite a bit	Very much
II36	My life is meaningful					
II36-B	How true was this before your illness?	☐ 2	☐ 2	☐ 3	☐ 4	☐ 5
II36-A	How true is this now, since your illness?	☐ 2	☐ 2	☐ 3	☐ 4	☐ 5

		Not at all	A little bit	Somewhat	Quite a bit	Very much
II37	I appreciate life					
II37-B	How true was this before your illness?	☐ 2	☐ 2	☐ 3	☐ 4	☐ 5
II37-A	How true is this now, since your illness?	☐ 2	☐ 2	☐ 3	☐ 4	☐ 5

		Not at all	A little bit	Somewhat	Quite a bit	Very much
II39	I have a sense of purpose in life					
II39-B	How true was this before your illness?	☐ 2	☐ 2	☐ 3	☐ 4	☐ 5
II39-A	How true is this now, since your illness?	☐ 2	☐ 2	☐ 3	☐ 4	☐ 5

		Not at all	A little bit	Somewhat	Quite a bit	Very much
II40	I feel peaceful					
II40-B	How true was this before your illness?	☐ 2	☐ 2	☐ 3	☐ 4	☐ 5
II40-A	How true is this now, since your illness?	☐ 2	☐ 2	☐ 3	☐ 4	☐ 5

		Not at all	A little bit	Somewhat	Quite a bit	Very much
II43	I have a sense of peace					
II43-B	How true was this before your illness?	☐ 2	☐ 2	☐ 3	☐ 4	☐ 5
II43-A	How true is this now, since your illness?	☐ 2	☐ 2	☐ 3	☐ 4	☐ 5

		Not at all	A little bit	Somewhat	Quite a bit	Very much
II46	I see what is really important in my life					
II46-B	How true was this before your illness?	☐ 2	☐ 2	☐ 3	☐ 4	☐ 5

© 2008-2016 PROMIS Health Organization and PROMIS Cooperative Group

II46-A	How true is this now, <u>since your illness</u>?	☐ 2	☐ 2	☐ 3	☐ 4	☐ 5

Physical Function

Please respond to each item by marking one box per row.

		Without any difficulty	With a little difficulty	With some difficulty	With much difficulty	Unable to do
PFA8	Are you able to move a chair from one room to another?......................................	☐ 5	☐ 4	☐ 3	☐ 2	☐ 1
PFA9	Are you able to bend down and pick up clothing from the floor?.............................	☐ 5	☐ 4	☐ 3	☐ 2	☐ 1
PFA10	Are you able to stand for one hour?	☐ 5	☐ 4	☐ 3	☐ 2	☐ 1
PFA11	Are you able to do chores such as vacuuming or yard work?..........................	☐ 5	☐ 4	☐ 3	☐ 2	☐ 1
PFA12	Are you able to push open a heavy door? ...	☐ 5	☐ 4	☐ 3	☐ 2	☐ 1
PFA13	Are you able to exercise for an hour?	☐ 5	☐ 4	☐ 3	☐ 2	☐ 1
PFA14r1	Are you able to carry a heavy object (over 10 pounds /5 kg)?	☐ 5	☐ 4	☐ 3	☐ 2	☐ 1
PFA15	Are you able to stand up from an armless straight chair?	☐ 5	☐ 4	☐ 3	☐ 2	☐ 1
PFA16r1	Are you able to dress yourself, including tying shoelaces and buttoning your clothes?	☐ 5	☐ 4	☐ 3	☐ 2	☐ 1
PFA17	Are you able to reach into a high cupboard?	☐ 5	☐ 4	☐ 3	☐ 2	☐ 1

© 2008-2016 PROMIS Health Organization and PROMIS Cooperative Group

PFA18	Are you able to use a hammer to pound a nail?	☐ 5	☐ 4	☐ 3	☐ 2	☐ 1

© 2008-2016 PROMIS Health Organization and PROMIS Cooperative Group

		Without any difficulty	With a little difficulty	With some difficulty	With much difficulty	Unable to do
PFA19r1	Are you able to run or jog for two miles (3 km)?..	☐ 5	☐ 4	☐ 3	☐ 2	☐ 1
PFA20	Are you able to cut your food using eating utensils?	☐ 5	☐ 4	☐ 3	☐ 2	☐ 1
PFA21	Are you able to go up and down stairs at a normal pace?	☐ 5	☐ 4	☐ 3	☐ 2	☐ 1
PFA23	Are you able to go for a walk of at least 15 minutes?	☐ 5	☐ 4	☐ 3	☐ 2	☐ 1
PFA27	Are you able to run on uneven ground?	☐ 5	☐ 4	☐ 3	☐ 2	☐ 1
PFA28	Are you able to open a can with a hand can opener?..	☐ 5	☐ 4	☐ 3	☐ 2	☐ 1
PFA29r1	Are you able to pull heavy objects (10 pounds/ 5 kg) towards yourself?	☐ 5	☐ 4	☐ 3	☐ 2	☐ 1
PFA30	Are you able to step up and down curbs?...	☐ 5	☐ 4	☐ 3	☐ 2	☐ 1
PFA31r1	Are you able to get up from the floor from lying on your back without help?	☐ 5	☐ 4	☐ 3	☐ 2	☐ 1
PFA32	Are you able to stand with your knees straight?	☐ 5	☐ 4	☐ 3	☐ 2	☐ 1
PFA33	Are you able to exercise hard for half an hour?	☐ 5	☐ 4	☐ 3	☐ 2	☐ 1
PFA34	Are you able to wash your back?	☐ 5	☐ 4	☐ 3	☐ 2	☐ 1

© 2008-2016 PROMIS Health Organization and PROMIS Cooperative Group

		Without any difficulty	With a little difficulty	With some difficulty	With much difficulty	Unable to do
PFA35	Are you able to open and close a zipper?..	☐ 5	☐ 4	☐ 3	☐ 2	☐ 1
PFA36	Are you able to put on and take off a coat or jacket?	☐ 5	☐ 4	☐ 3	☐ 2	☐ 1
PFA37	Are you able to stand for short periods of time?	☐ 5	☐ 4	☐ 3	☐ 2	☐ 1
PFA38	Are you able to dry your back with a towel?	☐ 5	☐ 4	☐ 3	☐ 2	☐ 1
PFA39r1	Are you able to run at a fast pace for two miles (3 km)?...	☐ 5	☐ 4	☐ 3	☐ 2	☐ 1
PFA40	Are you able to turn a key in a lock?.........	☐ 5	☐ 4	☐ 3	☐ 2	☐ 1
PFA41	Are you able to squat and get up?	☐ 5	☐ 4	☐ 3	☐ 2	☐ 1
PFA42	Are you able to carry a laundry basket up a flight of stairs?..	☐ 5	☐ 4	☐ 3	☐ 2	☐ 1
PFA43r1	Are you able to write with a pen or pencil?	☐ 4	☐ 3	☐ 2	☐ 1	☐ 1
PFA44	Are you able to put on a shirt or blouse?...	☐ 5	☐ 4	☐ 3	☐ 2	☐ 1
PFA45	Are you able to get out of bed into a chair?	☐ 5	☐ 4	☐ 3	☐ 2	☐ 1
PFA47	Are you able to pull on trousers?	☐ 5	☐ 4	☐ 3	☐ 2	☐ 1

© 2008-2016 PROMIS Health Organization and PROMIS Cooperative Group

		Without any difficulty	With a little difficulty	With some difficulty	With much difficulty	Unable to do
PFA48	Are you able to peel fruit?	☐ 5	☐ 4	☐ 3	☐ 2	☐ 1
PFA49	Are you able to bend or twist your back?...	☐ 5	☐ 4	☐ 3	☐ 2	☐ 1
PFA50	Are you able to brush your teeth?	☐ 5	☐ 4	☐ 3	☐ 2	☐ 1
PFA51	Are you able to sit on the edge of a bed? .	☐ 5	☐ 4	☐ 3	☐ 2	☐ 1
PFA52	Are you able to tie your shoelaces?	☐ 5	☐ 4	☐ 3	☐ 2	☐ 1
PFA53	Are you able to run errands and shop?	☐ 5	☐ 4	☐ 3	☐ 2	☐ 1
PFA54	Are you able to button your shirt?.............	☐ 5	☐ 4	☐ 3	☐ 2	☐ 1
PFA55	Are you able to wash and dry your body?..	☐ 5	☐ 4	☐ 3	☐ 2	☐ 1
PFA56	Are you able to get in and out of a car?	☐ 5	☐ 4	☐ 3	☐ 2	☐ 1
PFB8r1	Are you able to carry two bags filled with groceries 100 yards (100 m)?	☐ 5	☐ 4	☐ 3	☐ 2	☐ 1
PFB9	Are you able to jump up and down?	☐ 5	☐ 4	☐ 3	☐ 2	☐ 1
PFB10	Are you able to climb up five steps?	☐ 5	☐ 4	☐ 3	☐ 2	☐ 1

© 2008-2016 PROMIS Health Organization and PROMIS Cooperative Group

		Without any difficulty	With a little difficulty	With some difficulty	With much difficulty	Unable to do
PFB11	Are you able to wash dishes, pots, and utensils by hand while standing at a sink?	☐ 5	☐ 4	☐ 3	☐ 2	☐ 1
PFB12	Are you able to make a bed, including spreading and tucking in bed sheets?	☐ 5	☐ 4	☐ 3	☐ 2	☐ 1
PFB13	Are you able to carry a shopping bag or briefcase?...	☐ 5	☐ 4	☐ 3	☐ 2	☐ 1
PFB14	Are you able to take a tub bath?	☐ 5	☐ 4	☐ 3	☐ 2	☐ 1
PFB15r1	Are you able to change the bulb in a table lamp?	☐ 3	☐ 2	☐ 1	☐ 1	☐ 1
PFB16r1	Are you able to press with your index finger (for example ringing a doorbell)? ...	☐ 4	☐ 3	☐ 2	☐ 1	☐ 1
PFB17	Are you able to put on and take off your socks?	☐ 5	☐ 4	☐ 3	☐ 2	☐ 1
PFB18	Are you able to shave your face or apply makeup?	☐ 5	☐ 4	☐ 3	☐ 2	☐ 1
PFB19r1	Are you able to squeeze a new tube of toothpaste?..	☐ 4	☐ 3	☐ 2	☐ 1	☐ 1
PFB20r1	Are you able to cut a piece of paper with scissors?..	☐ 4	☐ 3	☐ 2	☐ 1	☐ 1
PFB21r1	Are you able to pick up coins from a table top?	☐ 4	☐ 3	☐ 2	☐ 1	☐ 1
PFB22	Are you able to hold a plate full of food?...	☐ 5	☐ 4	☐ 3	☐ 2	☐ 1

© 2008-2016 PROMIS Health Organization and PROMIS Cooperative Group

		Without any difficulty	With a little difficulty	With some difficulty	With much difficulty	Unable to do
PFB23r1	Are you able to pour liquid from a bottle into a glass?	☐ 4	☐ 3	☐ 2	☐ 1	☐ 1
PFB24	Are you able to run a short distance, such as to catch a bus?.......................................	☐ 5	☐ 4	☐ 3	☐ 2	☐ 1
PFB25	Are you able to push open a door after turning the knob?......................................	☐ 5	☐ 4	☐ 3	☐ 2	☐ 1
PFB26	Are you able to shampoo your hair?	☐ 5	☐ 4	☐ 3	☐ 2	☐ 1
PFB27	Are you able to tie a knot or a bow?..........	☐ 5	☐ 4	☐ 3	☐ 2	☐ 1
PFB28r1	Are you able to lift 10 pounds (5 kg) above your shoulder?	☐ 5	☐ 4	☐ 3	☐ 2	☐ 1
PFB29r1	Are you able to lift a full cup or glass to your mouth?...	☐ 4	☐ 3	☐ 2	☐ 1	☐ 1
PFB30	Are you able to open a new milk carton? ...	☐ 5	☐ 4	☐ 3	☐ 2	☐ 1
PFB31r1	Are you able to open car doors?	☐ 4	☐ 3	☐ 2	☐ 1	☐ 1
PFB32	Are you able to stand unsupported for 10 minutes?	☐ 5	☐ 4	☐ 3	☐ 2	☐ 1
PFB33	Are you able to remove something from your back pocket?.....................................	☐ 5	☐ 4	☐ 3	☐ 2	☐ 1
PFB34	Are you able to change a light bulb overhead?	☐ 5	☐ 4	☐ 3	☐ 2	☐ 1

© 2008-2016 PROMIS Health Organization and PROMIS Cooperative Group

		Without any difficulty	With a little difficulty	With some difficulty	With much difficulty	Unable to do
PFB36	Are you able to put on a pullover sweater?	☐ 5	☐ 4	☐ 3	☐ 2	☐ 1
PFB37r1	Are you able to turn faucets on and off?	☐ 4	☐ 3	☐ 2	☐ 1	☐ 1
PFB39r1	Are you able to reach and get down a 5 pound (2 kg) object from above your head?..	☐ 5	☐ 4	☐ 3	☐ 2	☐ 1
PFB40	Are you able to stand up on tiptoes?	☐ 5	☐ 4	☐ 3	☐ 2	☐ 1
PFB41	Are you able to trim your fingernails?	☐ 5	☐ 4	☐ 3	☐ 2	☐ 1
PFB42	Are you able to stand unsupported for 30 minutes?	☐ 5	☐ 4	☐ 3	☐ 2	☐ 1
PFB56r1	Are you able to lift one pound (0.5 kg) to shoulder level without bending your elbow?	☐ 5	☐ 4	☐ 3	☐ 2	☐ 1
PFC6r1	Are you able to walk a block (about 100 m) on flat ground?....................................	☐ 5	☐ 4	☐ 3	☐ 2	☐ 1
PFC7r1	Are you able to run five miles (8 km)?	☐ 5	☐ 4	☐ 3	☐ 2	☐ 1
PFC13r1	Are you able to run 100 yards (100 m)?.....	☐ 5	☐ 4	☐ 3	☐ 2	☐ 1
PFC21	Are you able to run on even ground?	☐ 5	☐ 4	☐ 3	☐ 2	☐ 1

© 2008-2016 PROMIS Health Organization and PROMIS Cooperative Group

		Without any difficulty	With a little difficulty	With some difficulty	With much difficulty	Unable to do
PFC29	Are you able to walk up and down two steps?	☐ 5	☐ 4	☐ 3	☐ 2	☐ 1
PFC30	Are you able to carry a suitcase up a flight of stairs?	☐ 5	☐ 4	☐ 3	☐ 2	☐ 1
PFC31	Are you able to reach into a low cupboard?	☐ 5	☐ 4	☐ 3	☐ 2	☐ 1
PFC32	Are you able to climb up 5 flights of stairs?..	☐ 5	☐ 4	☐ 3	☐ 2	☐ 1
PFC33r1	Are you able to run ten miles (16 km)?.....	☐ 5	☐ 4	☐ 3	☐ 2	☐ 1
PFC38	Are you able to walk at a normal speed?...	☐ 5	☐ 4	☐ 3	☐ 2	☐ 1
PFC39	Are you able to stand without losing your balance for several minutes?	☐ 5	☐ 4	☐ 3	☐ 2	☐ 1
PFC40	Are you able to kneel on the floor?	☐ 5	☐ 4	☐ 3	☐ 2	☐ 1
PFC41	Are you able to sit down in and stand up from a low, soft couch?	☐ 5	☐ 4	☐ 3	☐ 2	☐ 1
PFC42	Are you able to open a tight or new jar? ...	☐ 5	☐ 4	☐ 3	☐ 2	☐ 1
PFC43	Are you able to do use your hands, such as for turning faucets, using kitchen gadgets, or sewing?	☐ 5	☐ 4	☐ 3	☐ 2	☐ 1
PFC45r1	Are you able to sit on and get up from the toilet?	☐ 5	☐ 4	☐ 3	☐ 2	☐ 1

© 2008-2016 PROMIS Health Organization and PROMIS Cooperative Group

		Without any difficulty	With a little difficulty	With some difficulty	With much difficulty	Unable to do
PFC46	Are you able to transfer from a bed to a chair and back?	☐ 5	☐ 4	☐ 3	☐ 2	☐ 1
PFC47	Are you able to be out of bed most of the day?	☐ 5	☐ 4	☐ 3	☐ 2	☐ 1
PFC48	Are you able to carry household items, such as heavy boxes or furniture, up a flight of stairs?..	☐ 5	☐ 4	☐ 3	☐ 2	☐ 1
PFC49	Are you able to water a house plant?	☐ 5	☐ 4	☐ 3	☐ 2	☐ 1
PFC51	Are you able to wipe yourself after using the toilet?	☐ 5	☐ 4	☐ 3	☐ 2	☐ 1
PFC52	Are you able to turn from side to side in bed	☐ 5	☐ 4	☐ 3	☐ 2	☐ 1
PFC53	Are you able to get in and out of bed?	☐ 5	☐ 4	☐ 3	☐ 2	☐ 1
PFM1	Are you able to dig a 2-foot (1/2 m) deep hole in the dirt with a shovel?	☐ 5	☐ 4	☐ 3	☐ 2	☐ 1
PFM2	Are you able to lift a heavy painting or picture to hang on your wall above eye- level?	☐ 5	☐ 4	☐ 3	☐ 2	☐ 1
PFM3	Are you able to paint the walls of a room with a brush or roller for 2 hours without stopping to rest?	☐ 5	☐ 4	☐ 3	☐ 2	☐ 1
PFM4	Are you able to row a boat for 30 minutes without stopping to rest?	☐ 5	☐ 4	☐ 3	☐ 2	☐ 1

© 2008-2016 PROMIS Health Organization and PROMIS Cooperative Group

		Without any difficulty	With a little difficulty	With some difficulty	With much difficulty	Unable to do
PFM6	Are you able to hand wash and wax a car for 2 hours without stopping to rest?..........	☐ 5	☐ 4	☐ 3	☐ 2	☐ 1
PFM7	Are you able to complete 5 push-ups without stopping?	☐ 5	☐ 4	☐ 3	☐ 2	☐ 1
PFM9	Are you able to rake leaves or sweep for an hour without stopping to rest?...............	☐ 5	☐ 4	☐ 3	☐ 2	☐ 1
PFM10	Are you able to do a pull-up?	☐ 5	☐ 4	☐ 3	☐ 2	☐ 1
PFM12	Are you able to lift a heavy object (20 lbs/10 kg) above your head?......................	☐ 5	☐ 4	☐ 3	☐ 2	☐ 1
PFM15	Are you able to hit the backboard with a basketball from the free-throw line (13 ft/4 m)?..	☐ 5	☐ 4	☐ 3	☐ 2	☐ 1
PFM16	Are you able to pass a 20-pound (10 kg) turkey or ham to other people at the table?	☐ 5	☐ 4	☐ 3	☐ 2	☐ 1
PFM17	Are you able to remove a heavy suitcase (50 lbs/25 kg) from an overhead bin on an airplane or bus?	☐ 5	☐ 4	☐ 3	☐ 2	☐ 1
PFM18	Are you able to continuously swing a baseball bat or tennis racket back and forth for 5 minutes?	☐ 5	☐ 4	☐ 3	☐ 2	☐ 1
PFM19	Are you able to complete 10 sit-ups without stopping?	☐ 5	☐ 4	☐ 3	☐ 2	☐ 1
PFM21	Are you able to climb the stairs of a 10- story building without stopping?................	☐ 5	☐ 4	☐ 3	☐ 2	☐ 1

© 2008-2016 PROMIS Health Organization and PROMIS Cooperative Group

		Without any difficulty	With a little difficulty	With some difficulty	With much difficulty	Unable to do
PFM23	Are you able to walk briskly for 20 minutes without stopping to rest?.............	☐ 5	☐ 4	☐ 3	☐ 2	☐ 1
PFM25	Are you able to come to a complete stop while running?..	☐ 5	☐ 4	☐ 3	☐ 2	☐ 1
PFM26	Are you able to make sharp turns while running fast?	☐ 5	☐ 4	☐ 3	☐ 2	☐ 1
PFM27	Are you able to jump rope for 10 minutes without stopping?	☐ 5	☐ 4	☐ 3	☐ 2	☐ 1
PFM28	Are you able to jump over an object that is 1 foot (30 cm) tall?	☐ 5	☐ 4	☐ 3	☐ 2	☐ 1
PFM29	Are you able to jump over a puddle that is 3 feet (1 m) wide?....................................	☐ 5	☐ 4	☐ 3	☐ 2	☐ 1
PFM32	Are you able to jump 2 feet (60 cm) high?	☐ 5	☐ 4	☐ 3	☐ 2	☐ 1
PFM33	Are you able to walk across a balance beam?...	☐ 5	☐ 4	☐ 3	☐ 2	☐ 1
PFM34	Are you able to stand on one foot with your eyes closed for 30 seconds?	☐ 5	☐ 4	☐ 3	☐ 2	☐ 1
PFM35	Are you able to walk in a straight line putting one foot in front of the other (heel to toe) for 5 yards (5 m)?..........................	☐ 5	☐ 4	☐ 3	☐ 2	☐ 1
PFM36	Are you able to put your hands flat on the floor with both feet flat on the ground?.....	☐ 5	☐ 4	☐ 3	☐ 2	☐ 1
PFM37	Are you able to carry a large baby (15 lbs/7 kg) out of the house to a car or taxi?	☐ 5	☐ 4	☐ 3	☐ 2	☐ 1

© 2008-2016 PROMIS Health Organization and PROMIS Cooperative Group

		Without any difficulty	With a little difficulty	With some difficulty	With much difficulty	Unable to do
PFM38	Are you able to lift and load one 50-pound (25 kg) bag of sand into a car?	☐ 5	☐ 4	☐ 3	☐ 2	☐ 1
PFM40	Are you able to climb a 6-foot (2 m) ladder?	☐ 5	☐ 4	☐ 3	☐ 2	☐ 1
PFM43	Are you able to push an empty refrigerator forward 1 yard (1 m)?................................	☐ 5	☐ 4	☐ 3	☐ 2	☐ 1
PFM44	Are you able to carry a 50 lb (25 kg) bag of sand 25 yards (25 m)?	☐ 5	☐ 4	☐ 3	☐ 2	☐ 1
PFM46	Are you able to pull a sled or a wagon with two children (total 100 lbs/50 kg) for 100 yards (100 m)?...................................	☐ 5	☐ 4	☐ 3	☐ 2	☐ 1
PFM49	Are you able to stand up from a push-up position five times quickly?	☐ 5	☐ 4	☐ 3	☐ 2	☐ 1
PFM51	Are you able to swim laps for 30 minutes at a moderate pace?	☐ 5	☐ 4	☐ 3	☐ 2	☐ 1
PFM53	Are you able to dance energetically for an hour?	☐ 5	☐ 4	☐ 3	☐ 2	☐ 1

		Not at all	Very little	Somewhat	Quite a lot	Cannot do
PFA1	Does your health now limit you in doing vigorous activities, such as running, lifting heavy objects, participating in strenuous sports?...	☐ 5	☐ 4	☐ 3	☐ 2	☐ 1
PFA2	Does your health now limit you in exercising regularly?	☐ 5	☐ 4	☐ 3	☐ 2	☐ 1
PFA3	Does your health now limit you in bending, kneeling, or stooping?	☐ 5	☐ 4	☐ 3	☐ 2	☐ 1

© 2008-2016 PROMIS Health Organization and PROMIS Cooperative Group

		Not at all	Very little	Somewhat	Quite a lot	Cannot do
PFA4	Does your health now limit you in doing heavy work around the house like scrubbing floors, or lifting or moving heavy furniture?.......................................	☐ 5	☐ 4	☐ 3	☐ 2	☐ 1
PFA5	Does your health now limit you in lifting or carrying groceries?	☐ 5	☐ 4	☐ 3	☐ 2	☐ 1
PFA6	Does your health now limit you in bathing or dressing yourself?	☐ 5	☐ 4	☐ 3	☐ 2	☐ 1
PFB1	Does your health now limit you in doing moderate work around the house like vacuuming, sweeping floors or carrying in groceries?..	☐ 5	☐ 4	☐ 3	☐ 2	☐ 1
PFB3	Does your health now limit you in putting a trash bag outside?	☐ 5	☐ 4	☐ 3	☐ 2	☐ 1
PFB4	Does your health now limit you in dancing for half an hour?..........................	☐ 5	☐ 4	☐ 3	☐ 2	☐ 1
PFB5r1	Does your health now limit you in hiking a couple of miles (3 km) on uneven surfaces, including hills?..........................	☐ 5	☐ 4	☐ 3	☐ 2	☐ 1
PFB7	Does your health now limit you in doing strenuous activities such as backpacking, skiing, playing tennis, bicycling or jogging?	☐ 5	☐ 4	☐ 3	☐ 2	☐ 1
PFB43	Does your health now limit you in taking care of your personal needs (dress, comb hair, toilet, eat, bathe)?.............................	☐ 5	☐ 4	☐ 3	☐ 2	☐ 1
PFB44	Does your health now limit you in doing moderate activities, such as moving a table, pushing a vacuum cleaner, bowling, or playing golf?	☐ 5	☐ 4	☐ 3	☐ 2	☐ 1

© 2008-2016 PROMIS Health Organization and PROMIS Cooperative Group

		Not at all	Very little	Somewhat	Quite a lot	Cannot do
PFB45	Does your health now limit you in taking part in any sports (swimming, bowling, and so forth)?...	☐ 5	☐ 4	☐ 3	☐ 2	☐ 1
PFB48	Does your health now limit you in taking a shower?...	☐ 5	☐ 4	☐ 3	☐ 2	☐ 1
PFB49	Does your health now limit you in going for a short walk (less than 15 minutes)?....	☐ 5	☐ 4	☐ 3	☐ 2	☐ 1
PFB51	Does your health now limit you in participating in active sports such as swimming, tennis, or basketball?	☐ 5	☐ 4	☐ 3	☐ 2	☐ 1
PFB54	Does your health now limit you in going OUTSIDE the home, for example to shop or visit a doctor's office?	☐ 5	☐ 4	☐ 3	☐ 2	☐ 1
PFC8	Does your health now limit you in opening a previously opened jar?.............	☐ 5	☐ 4	☐ 3	☐ 2	☐ 1
PFC10	Does your health now limit you in climbing several flights of stairs?.............	☐ 5	☐ 4	☐ 3	☐ 2	☐ 1
PFC11	Does your health now limit you in doing yard work like raking leaves, weeding, or pushing a lawn mower?	☐ 5	☐ 4	☐ 3	☐ 2	☐ 1
PFC12	Does your health now limit you in doing two hours of physical labor?	☐ 5	☐ 4	☐ 3	☐ 2	☐ 1
PFC35	Does your health now limit you in doing eight hours of physical labor?	☐ 5	☐ 4	☐ 3	☐ 2	☐ 1
PFC36r1	Does your health now limit you in walking more than a mile (1.6 km)?..........	☐ 5	☐ 4	☐ 3	☐ 2	☐ 1

© 2008-2016 PROMIS Health Organization and PROMIS Cooperative Group

		Not at all	Very little	Somewhat	Quite a lot	Cannot do
PFC37	Does your health now limit you in climbing one flight of stairs?....................	☐ 5	☐ 4	☐ 3	☐ 2	☐ 1
PFC54	Does your health now limit you in getting in and out of the bathtub?	☐ 5	☐ 4	☐ 3	☐ 2	☐ 1
PFC56	Does your health now limit you in walking about the house?.........................	☐ 5	☐ 4	☐ 3	☐ 2	☐ 1

		No difficulty at all	A little bit of difficulty	Some difficulty	A lot of difficulty	Can't do because of health
PFB50	How much difficulty do you have doing your daily physical activities, because of your health?	☐ 5	☐ 4	☐ 3	☐ 2	☐ 1

		Completely	Mostly	Moderately	A little	Not at all
Global06	To what extent are you able to carry out your everyday physical activities such as walking, climbing stairs, carrying groceries, or moving a chair?	☐ 5	☐ 4	☐ 3	☐ 2	☐ 1

Cognitive Function

Please respond to each question or statement by marking one box per row.

In the past 7 days...

		Never	Rarely (Once)	Sometimes (Two or three times)	Often (About once a day)	Very often (Several times a day)
PC1r	I have had trouble forming thoughts........	☐ 5	☐ 4	☐ 3	☐ 2	☐ 1
PC2r	My thinking has been slow......................	☐ 5	☐ 4	☐ 3	☐ 2	☐ 1
PC3r	My thinking has been foggy....................	☐ 5	☐ 4	☐ 3	☐ 2	☐ 1

PC5r	I have had trouble adding or subtracting numbers in my head	☐ 5	☐ 4	☐ 3	☐ 2	☐ 1
PC7r	I have made mistakes when writing down phone numbers	☐ 5	☐ 4	☐ 3	☐ 2	☐ 1
PC8r	I have had trouble concentrating	☐ 5	☐ 4	☐ 3	☐ 2	☐ 1
PC10r	I have had trouble finding my way to a familiar place...	☐ 5	☐ 4	☐ 3	☐ 2	☐ 1
PC11r	I have had trouble remembering where I put things, like my keys or my wallet	☐ 5	☐ 4	☐ 3	☐ 2	☐ 1
PC12r	I have had trouble remembering whether I did things I was supposed to do, like taking a medicine or buying something I needed...	☐ 5	☐ 4	☐ 3	☐ 2	☐ 1

In the past 7 days...

		Never	Rarely (Once)	Sometimes (Two or three times)	Often (About once a day)	Very often (Several times a day)
PC13r	I have had trouble remembering new information, like phone numbers or simple instructions	☐ 5	☐ 4	☐ 3	☐ 2	☐ 1
PC14r	I have had trouble recalling the name of an object while talking to someone..............	☐ 5	☐ 4	☐ 3	☐ 2	☐ 1
PC18r	I have had trouble speaking fluently	☐ 5	☐ 4	☐ 3	☐ 2	☐ 1
PC21r	I have walked into a room and forgotten what I meant to get or do there................	☐ 5	☐ 4	☐ 3	☐ 2	☐ 1
PC22r	I have needed medical instructions repeated because I could not keep them straight	☐ 5	☐ 4	☐ 3	☐ 2	☐ 1
PC25r	I have had to work really hard to pay attention or I would make a mistake	☐ 5	☐ 4	☐ 3	☐ 2	☐ 1
PC26r	I have forgotten names of people soon after being introduced..............................	☐ 5	☐ 4	☐ 3	☐ 2	☐ 1
PC28r	My reactions in everyday situations have been slow..	☐ 5	☐ 4	☐ 3	☐ 2	☐ 1
PC30r	Other people have told me I seemed to have trouble remembering information....	☐ 5	☐ 4	☐ 3	☐ 2	☐ 1
PC35r	It has seemed like my brain was not working as well as usual..........................	☐ 5	☐ 4	☐ 3	☐ 2	☐ 1
PC36r	I have had to work harder than usual to keep track of what I was doing................	☐ 5	☐ 4	☐ 3	☐ 2	☐ 1

In the past 7 days...

		Never	Rarely (Once)	Sometimes (Two or three times)	Often (About once a day)	Very often (Several times a day)
PC37r	My thinking has been slower than usual ..	☐ 5	☐ 4	☐ 3	☐ 2	☐ 1
PC38r	I have had to work harder than usual to express myself clearly	☐ 5	☐ 4	☐ 3	☐ 2	☐ 1
PC39r	I have had more problems conversing with others	☐ 5	☐ 4	☐ 3	☐ 2	☐ 1
PC40r	I have had to use written lists more often than usual so I would not forget things	☐ 5	☐ 4	☐ 3	☐ 2	☐ 1
PC41r	I have had trouble keeping track of what I was doing when interrupted	☐ 5	☐ 4	☐ 3	☐ 2	☐ 1
PC42r	I have had trouble shifting back and forth between different activities that require thinking...	☐ 5	☐ 4	☐ 3	☐ 2	☐ 1
PC48r	I have hidden my problems with memory, concentration, or making mental mistakes so that others would not notice................	☐ 5	☐ 4	☐ 3	☐ 2	☐ 1
PC49r	I have been upset about my problems with memory, concentration, or making mental mistakes	☐ 5	☐ 4	☐ 3	☐ 2	☐ 1
PC50r	My problems with memory, concentration, or making mental mistakes have interfered with my ability to work	☐ 5	☐ 4	☐ 3	☐ 2	☐ 1
PC51r	My problems with memory, concentration, or making mental mistakes have interfered with my ability to do things I enjoy	☐ 5	☐ 4	☐ 3	☐ 2	☐ 1

In the past 7 days...

PC53r	My problems with memory, concentration, or making mental mistakes have interfered with the quality of my life	☐ 5	☐ 4	☐ 3	☐ 2	☐ 1
PC-CaPS25r	I have had difficulty multi-tasking	☐ 5	☐ 4	☐ 3	☐ 2	☐ 1

Dyspnea Time Extension

Please respond to each question or statement by marking one box per row.
Considering your shortness of breath over the past 7 days, rate the amount of time it has taken you to complete the following activities to your satisfaction.
In the past 7 days…

		Less time	The same amount of time	More time	Does not apply
DYSTE001	How much time did it take you to use the toilet without help compared to 3 months ago?..	☐ -1	☐ 0	☐ 1	☐ 0
DYSTE002	How much time did it take you to wash your face completely compared to 3 months ago?..	☐ -1	☐ 0	☐ 1	☐ 0
DYSTE003	How much time did it take you to make your bed completely compared to 3 months ago?..	☐ -1	☐ 0	☐ 1	☐ 0
DYSTE004	How much time did it take you to carry less than 5 lbs (about 2 kg) compared to 3 months ago?..	☐ -1	☐ 0	☐ 1	☐ 0
DYSTE005	How much time did it take you to walk up 10 stairs (1 flight) without stopping compared to 3 months ago?..	☐ -1	☐ 0	☐ 1	☐ 0
DYSTE006	How much time did it take you to carry something weighing 10-20 pounds (like a large bag of groceries) from one room to another compared to 3 months ago?..........	☐ -1	☐ 0	☐ 1	☐ 0
DYSTE007	How much time has it taken you to do your usual activities in general, compared to 3 months ago?	☐ -1	☐ 0	☐ 1	☐ 0

Global Health – Mental 2a

Please respond to each question or statement by marking one box per row.

		Excellent	Very good	Good	Fair	Poor
Global04	In general, how would you rate your mental health, including your mood and your ability to think?	☐ 5	☐ 4	☐ 3	☐ 2	☐ 1
Global05	In general, how would you rate your satisfaction with your social activities and relationships?..	☐ 5	☐ 4	☐ 3	☐ 2	☐ 1

Psychosocial Illness Impact-Positive–Short Form 8a
Please respond to each question or statement by marking one box per row.

Thinking about how your illness has affected you, please rate how true these statements were of you before your illness, and again now, since your illness.

		Not at all	A little bit	Somewhat	Quite a bit	Very much
II2	I am comfortable with who I am					
II2.b	How true was this before your illness?	☐ 2	☐ 2	☐ 3	☐ 4	☐ 5
II2.a	How true is this now, since your illness?	☐ 2	☐ 2	☐ 3	☐ 4	☐ 5
II17	I realize who my real friends are					
II17.b	How true was this before your illness?	☐ 2	☐ 2	☐ 3	☐ 4	☐ 5
II17.a	How true is this now, since your illness?	☐ 2	☐ 2	☐ 3	☐ 4	☐ 5
II27	I can adjust to things I cannot change					
II27.b	How true was this before your illness?	☐ 2	☐ 2	☐ 3	☐ 4	☐ 5
II27.a	How true is this now, since your illness?	☐ 2	☐ 2	☐ 3	☐ 4	☐ 5
II36	My life is meaningful					
II36.b	How true was this before your illness?	☐ 2	☐ 2	☐ 3	☐ 4	☐ 5

		Not at all	A little bit	Somewhat	Quite a bit	Very much
II36.a	How true is this now, since your illness?	☐ 2	☐ 2	☐ 3	☐ 4	☐ 5

II7	I am an optimistic person	Not at all	A little bit	Somewhat	Quite a bit	Very much
II7.b	How true was this before your illness?	☐ 2	☐ 2	☐ 3	☐ 4	☐ 5
II7.a	How true is this now, since your illness?	☐ 2	☐ 2	☐ 3	☐ 4	☐ 5

Thinking about how your illness has affected you, please rate how true these statements were of you before your illness, and again now, since your illness.

II15	My relationships are meaningful	Not at all	A little bit	Somewhat	Quite a bit	Very much
II15.b	How true was this before your illness?	☐ 2	☐ 2	☐ 3	☐ 4	☐ 5
II15.a	How true is this now, since your illness?	☐ 2	☐ 2	☐ 3	☐ 4	☐ 5

II32	I look at things in a positive way	Not at all	A little bit	Somewhat	Quite a bit	Very much
II32.b	How true was this before your illness?	☐ 2	☐ 2	☐ 3	☐ 4	☐ 5
II32.a	How true is this now, since your illness?	☐ 2	☐ 2	☐ 3	☐ 4	☐ 5

II35	I can appreciate each day fully	Not at all	A little bit	Somewhat	Quite a bit	Very much
II35.b	How true was this before your illness?	☐ 2	☐ 2	☐ 3	☐ 4	☐ 5
II35.a	How true is this now, since your illness?	☐ 2	☐ 2	☐ 3	☐ 4	☐ 5

REFERENCES

1. Bastard P, Rosen LB, Zhang Q, et al. Autoantibodies against type I IFNs in patients with life-threatening COVID-19. *Science* 2020; **370**(6515).
2. Team IMW, Committee INS. Immunophenotyping assessment in a COVID-19 cohort (IMPACC): A prospective longitudinal study. *Science immunology* 2021; **6**(62): eabf3733.
3. Evans AM, DeHaven CD, Barrett T, Mitchell M, Milgram E. Integrated, nontargeted ultrahigh performance liquid chromatography/electrospray ionization tandem mass spectrometry platform for the identification and relative quantification of the small-molecule complement of biological systems. *Anal Chem* 2009; **81**(16): 6656-67.
4. Ford L, Kennedy AD, Goodman KD, et al. Precision of a Clinical Metabolomics Profiling Platform for Use in the Identification of Inborn Errors of Metabolism. *J Appl Lab Med* 2020; **5**(2): 342-56.
