# Supplementary material for: Features of acute COVID-19 associated with post-acute sequelae of SARS-CoV-2 phenotypes: results from the IMPACC study
Source: Nat Commun. 2024 Jan 3;15:216. doi: 10.1038/s41467-023-44090-5 (PMC10764789; doi:10.1038/s41467-023-44090-5)
Supplement: Supplementary file 3 — Reporting Summary [file 41467_2023_44090_MOESM3_ESM.pdf]

nature portfolio

Corresponding author(s): Nadine Rouphael, MD

Last updated by author(s): Nov 13, 2023

Reporting Summary

Nature Portfolio wishes to improve the reproducibility of the work that we publish. This form provides structure for consistency and transparency in reporting. For further information on Nature Portfolio policies, see our Editorial Policies and the Editorial Policy Checklist.

Statistics

For all statistical analyses, confirm that the following items are present in the figure legend, table legend, main text, or Methods section.

n/a	Confirmed	
☐	☒	The exact sample size (*n*) for each experimental group/condition, given as a discrete number and unit of measurement
☐	☒	A statement on whether measurements were taken from distinct samples or whether the same sample was measured repeatedly
☐	☒	The statistical test(s) used AND whether they are one- or two-sided<br>*Only common tests should be described solely by name; describe more complex techniques in the Methods section.*
☐	☒	A description of all covariates tested
☐	☒	A description of any assumptions or corrections, such as tests of normality and adjustment for multiple comparisons
☐	☒	A full description of the statistical parameters including central tendency (e.g. means) or other basic estimates (e.g. regression coefficient) AND variation (e.g. standard deviation) or associated estimates of uncertainty (e.g. confidence intervals)
☐	☒	For null hypothesis testing, the test statistic (e.g. *F*, *t*, *r*) with confidence intervals, effect sizes, degrees of freedom and *P* value noted<br>*Give P values as exact values whenever suitable.*
☒	☐	For Bayesian analysis, information on the choice of priors and Markov chain Monte Carlo settings
☒	☐	For hierarchical and complex designs, identification of the appropriate level for tests and full reporting of outcomes
☒	☐	Estimates of effect sizes (e.g. Cohen's *d*, Pearson's *r*), indicating how they were calculated

Our web collection on statistics for biologists contains articles on many of the points above.

Software and code

Policy information about availability of computer code

Data collection	Patient-reported data were collected using a mobile application developed by MyOwnMed, Inc. Along with the mobile application, an administrative portal was developed to collect information by study personnel during site visits or via telephone interview by a study coordinator to ensure real-time electronic data capture.
Data analysis	Data were analyzed using R statistical software version 4.2.1. Packages used are noted in the text. Flowjo was used for immunologic analysis version 10.9.0. These were added to the main paper and supplemental materials as well as in the reporting summary.<br>The BitBucket web link is HYPERLINK "https://bitbucket.org/kleinstein/impacc-public-code"https://bitbucket.org/kleinstein/impacc-public-code/src/master/convalescent_manuscript/. This is listed in the main paper as well as reporting summary. We have dedicated sections for data availability and code availability.

For manuscripts utilizing custom algorithms or software that are central to the research but not yet described in published literature, software must be made available to editors and reviewers. We strongly encourage code deposition in a community repository (e.g. GitHub). See the Nature Portfolio guidelines for submitting code & software for further information.

Data

Policy information about availability of data

All manuscripts must include a data availability statement. This statement should provide the following information, where applicable:

- Accession codes, unique identifiers, or web links for publicly available datasets
- A description of any restrictions on data availability
- For clinical datasets or third party data, please ensure that the statement adheres to our policy

All data necessary to reproduce the analysis presented will be submitted to ImmPort and will be made available under access control to researchers conducting COVID-related research to comply with the requirements of samples gathered under the public health exception during the pandemic.

The IMPACC Data Sharing Plan is designed to enable the widest dissemination of data, while also protecting the privacy of the participants and the utility of the data by de-identifying and masking potentially sensitive data elements. All IMPACC data including those generated in this study have been deposited in the Immunology Database and Analysis Portal (ImmPort), a NIAID Division of Allergy, Immunology and Transplantation funded data repository under accession code SDY1760 (immport.org). All data are available under restricted access to comply with NIH Public data sharing policy for IRB-exempted public surveillance studies, Access can be obtained via AccessClinicalData@NIAID (https://accessclinicaldata.niaid.nih.gov/study-viewer/clinical_trials). Additional guidelines for access are outlined on ImmPort (https://docs.immport.org/home/impaccslides). The raw and processed assay data are available at ImmPort SDY1790. In addition, raw LC-MS data for metabolomics are submitted to Metabolights repository under accession number MTBLS850. The source data used in the figures are provided as a Source Data file.

This statement is provided in the reporting summary as well as the main paper under data availability section- reasons for controlled access are to comply with NIH Public data sharing policy for IRB-exempted public surveillance studies and are for an indefinite period of time

Research involving human participants, their data, or biological material

Policy information about studies with human participants or human data. See also policy information about sex, gender (identity/presentation), and sexual orientation and race, ethnicity and racism.

Reporting on sex and gender	Sex was collected based on self-reporting.<br>The findings do not apply to one sex only.<br>The analysis included 61% males and 39% females.
Reporting on race, ethnicity, or other socially relevant groupings	The IMPACC cohort was diverse, with 31% participants being Hispanic/Latinx, and 22% black/African American, reflecting communities disproportionately affected by COVID-19,12 and with wide geographic distribution within the USA. Race/ ethnicity are reported in table 1; race was categorized as white, black, or other/declined/unknown/missing. Ethnicity was defined as Non-Hispanic/Hispanic.
Population characteristics	The covariate-relevant population characteristics have been described in Table 1.<br>The population characteristics described are:<br>comorbidities<br>BMI<br>Number of comorbidities<br>Health status at the baseline visit<br>Baseline labs<br>Severity and utilization during acute hospitalization<br>Acute complications reported<br>Medications during acute hospitalization<br>Convalescent symptoms
Recruitment	Patients 18 years and older admitted to 20 US hospitals (affiliated with 15 academic institutions) were enrolled within 48 hours of hospital admission for COVID-19 infection. Only confirmed positive SARS-CoV-2 PCR and symptomatic cases attributable to COVID-19 infection were followed longitudinally. Computer based alerts based on positive PCR for SARS-CoV-2 or isolation orders for COVID-19 facilitated recruitment. Site specific recruitment practices varied based on staffing.<br>There were no self-selection biases or other biases in the recruitment process.
Ethics oversight	Office for Human Research Protections concurred that the study satisfied criteria for the public health surveillance exception, and the IMPACC study team sent the study protocol, and participant information sheet for review, and assessment to institutional review boards (IRBs) at participating institutions. Twelve institutions elected to conduct the study as public health surveillance, while 3 sites with prior IRB-approved biobanking protocols elected to integrate and conduct IMPACC under their institutional protocols (University of Texas at Austin, IRB 202-04-0117; University of California San Francisco, IRB 20-30497; Case Western Reserve University, IRB STUDY20200573) with informed consent requirements. When ICF was not obtained under the public health surveillance expemption, an information sheet was provided to the participants and they were allowed to opted out at any time.

Note that full information on the approval of the study protocol must also be provided in the manuscript.

Field-specific reporting

Please select the one below that is the best fit for your research. If you are not sure, read the appropriate sections before making your selection.

☒ Life sciences ☐ Behavioural & social sciences ☐ Ecological, evolutionary & environmental sciences

For a reference copy of the document with all sections, see nature.com/documents/nr-reporting-summary-flat.pdf

Life sciences study design

All studies must disclose on these points even when the disclosure is negative.

Sample size	No sample size calculation was performed. This study was designed as an observational surveillance cohort with primary scientific aims that were descriptive. This was a convenience sample limited by funding constraints. The sample size was selected to ensure no greater than +/-3.1% for 95% confidence intervals around estimates for binary endpoints.
Data exclusions	No data were excluded from the full survey cohort of n=589 participants. The full diagram of eligible participants included in this cohort was published previously (Ozonoff et al. https://doi.org/10.1016/j.ebiom.2022.104208)
Replication	Because this was a single observational cohort, there were no opportunities to replicate the analyses.
Randomization	Randomization is not relevant to this study. Participants were not allocated to experimental groups. The study design is a single prospective study.
Blinding	Blinding is not relevant to this study. Participants were not allocated to experimental groups. The study design is a single prospective observational cohort.

Reporting for specific materials, systems and methods

We require information from authors about some types of materials, experimental systems and methods used in many studies. Here, indicate whether each material, system or method listed is relevant to your study. If you are not sure if a list item applies to your research, read the appropriate section before selecting a response.

Materials & experimental systems

n/a	Involved in the study	
☐	☒	Antibodies
☒	☐	Eukaryotic cell lines
☒	☐	Palaeontology and archaeology
☒	☐	Animals and other organisms
☐	☒	Clinical data
☒	☐	Dual use research of concern
☒	☐	Plants

Methods

n/a	Involved in the study	
☒	☐	ChIP-seq
☐	☒	Flow cytometry
☒	☐	MRI-based neuroimaging

Antibodies

Antibodies used	All antibodies have been listed in the supplemental material.
Validation	All resources and reagents have been listed in the supplemental material. Literature on the assays is available in the following papers (PMID: 32398876, 32302069, 33142304). No formal antibody validation was performed.

Clinical data

Policy information about clinical studies

All manuscripts should comply with the ICMJE guidelines for publication of clinical research and a completed CONSORT checklist must be included with all submissions.

Clinical trial registration	NCT0438777
Study protocol	https://clinicaltrials.gov/ct2/show/NCT04378777
Data collection	The study design, including data collection sites and procedures, has been published in Science Immunology: https://doi.org/10.1126/sciimmunol.abf3733
Outcomes	Patient-reported outcomes (PROs) are defined and described in the manuscript text. Other clinical outcomes are described in previous publications:

https://doi.org.10.1016/j.ebiom.2022.104208
https://doi.org/10.1126/sciimmunol.abf3733

Plants

Seed stocks

Report on the source of all seed stocks or other plant material used. If applicable, state the seed stock centre and catalogue number. If plant specimens were collected from the field, describe the collection location, date and sampling procedures.

Novel plant genotypes

Describe the methods by which all novel plant genotypes were produced. This includes those generated by transgenic approaches, gene editing, chemical/radiation-based mutagenesis and hybridization. For transgenic lines, describe the transformation method, the number of independent lines analyzed and the generation upon which experiments were performed. For gene-edited lines, describe the editor used, the endogenous sequence targeted for editing, the targeting guide RNA sequence (if applicable) and how the editor was applied.

Authentication

Describe any authentication procedures for each seed stock used or novel genotype generated. Describe any experiments used to assess the effect of a mutation and, where applicable, how potential secondary effects (e.g. second site T-DNA insertions, mosiacism, off-target gene editing) were examined.

Flow Cytometry

Plots

Confirm that:

☒ The axis labels state the marker and fluorochrome used (e.g. CD4-FITC).

☒ The axis scales are clearly visible. Include numbers along axes only for bottom left plot of group (a 'group' is an analysis of identical markers).

☒ All plots are contour plots with outliers or pseudocolor plots.

☒ A numerical value for number of cells or percentage (with statistics) is provided.

Methodology

Sample preparation

Blood samples were collected at study time points and processed within 6 hours for multiparameter CyTOF2. Whole blood anticoagulated with EDTA (270 µl) was labeled at room temperature for 30 min with a 30-marker Maxpar Direct Immune Profiling Assay (Standard Biotools, South San Francisco, CA) panels, fixed and cryopreserved using PROT-1 proteomic stabilizer (SMART, Inc, Las Vegas, NV) and frozen at -80C. Batches of samples were randomized by study site, disease severity (mild/moderate versus severe), and age (younger versus older) and thawed, washed, and labeled with a supplemental cocktail of 14 additional antibodies to identify additional fixation-resistant cell phenotype determinants.

Instrument

Helios mass cytometer

Software

FlowJo software version 10.9.0.

Cell population abundance

Pooled samples were acquired until a total of 6 x 106 cell events had been collected, corresponding to an average target event number of 3 x105 events per original donor subsample.

Gating strategy

Describe the gating strategy used for all relevant experiments, specifying the preliminary FSC/SSC gates of the starting cell population, indicating where boundaries between "positive" and "negative" staining cell populations are defined.

☐ Tick this box to confirm that a figure exemplifying the gating strategy is provided in the Supplementary Information.
